# Author Correction: Targeting SWI/SNF ATPases in enhancer-addicted prostate cancer

**DOI:** 10.1038/s41586-024-07393-1

**Published:** 2024-04-22

**Authors:** Lanbo Xiao, Abhijit Parolia, Yuanyuan Qiao, Pushpinder Bawa, Sanjana Eyunni, Rahul Mannan, Sandra E. Carson, Yu Chang, Xiaoju Wang, Yuping Zhang, Josh N. Vo, Steven Kregel, Stephanie A. Simko, Andrew D. Delekta, Mustapha Jaber, Heng Zheng, Ingrid J. Apel, Lisa McMurry, Fengyun Su, Rui Wang, Sylvia Zelenka-Wang, Sanjita Sasmal, Leena Khare, Subhendu Mukherjee, Chandrasekhar Abbineni, Kiran Aithal, Mital S. Bhakta, Jay Ghurye, Xuhong Cao, Nora M. Navone, Alexey I. Nesvizhskii, Rohit Mehra, Ulka Vaishampayan, Marco Blanchette, Yuzhuo Wang, Susanta Samajdar, Murali Ramachandra, Arul M. Chinnaiyan

**Affiliations:** 1 Michigan Center for Translational Pathology, University of Michigan, Ann Arbor, MI USA; 2 Department of Pathology, University of Michigan, Ann Arbor, MI USA; 3 Molecular and Cellular Pathology Program, University of Michigan, Ann Arbor, MI USA; 4 Rogel Cancer Center, University of Michigan, Ann Arbor, MI USA; 5 Department of Computational Medicine and Bioinformatics, University of Michigan, Ann Arbor, MI USA; 6 Aurigene Discovery Technologies, Electronic City Phase II, Bangalore, India; 7 Dovetail Genomics, Scotts Valley, CA USA; 8 Howard Hughes Medical Institute, University of Michigan, Ann Arbor, MI USA; 9 Department of Genitourinary Medical Oncology and the David H. Koch Center for Applied Research of Genitourinary Cancers, The University of Texas MD Anderson Cancer Center, Houston, TX USA; 10 Department of Internal Medicine/Oncology, University of Michigan, Ann Arbor, MI USA; 11 Vancouver Prostate Centre, Vancouver, British Columbia Canada; 12 Department of Urologic Sciences, University of British Columbia, Vancouver, British Columbia V6T 1Z3 Canada; 13 Department of Urology, University of Michigan, Ann Arbor, MI USA

**Keywords:** Targeted therapies, Prostate cancer, Chromatin remodelling

Correction to: *Nature* 10.1038/s41586-021-04246-z Published online 22 December 2021

In the version of this article initially published, there was an error in column B, row 1 in Supplementary Table 1, where the unit incorrectly appeared as IC_50_ (μM) instead of IC_50_ (nM). In addition, we noticed that there was an image duplication of Extended Data Fig. 10h, where the image of SMARCA4/BRG1 IHC staining of kidney tissue from the enzalutamide group was inadvertently duplicated from the vehicle group. The conclusions of the paper remain unaffected. Supplementary Table 1 and Extended Data Fig. 10h have been updated to reflect these revisions.

