# Supplementary material for: Targeting SWI/SNF ATPases in enhancer-addicted prostate cancer
Source: Nature. 2021 Dec 22;601(7893):434–9. doi: 10.1038/s41586-021-04246-z (PMC8770127; doi:10.1038/s41586-021-04246-z)

nature portfolio

https://doi.org/10.1038/s41586-021-04246-z

Supplementary information

Targeting SWI/SNF ATPases in enhancer-addicted prostate cancer

In the format provided by the authors and unedited

SUPPLEMENTARY INFORMATION

Targeting SWI/SNF ATPases in enhancer-addicted prostate cancer

Lanbo Xiao[1,2,†], Abhijit Parolia[1,2,3,†], Yuanyuan Qiao[1,2,4,†], Pushpinder Bawa[1,2], Sanjana Eyunni[1,2,3], Rahul Mannan[1,2], Sandra E. Carson[1,2], Yu Chang[1,2], Xiaoju Wang[1,2,4], Yuping Zhang[1,2], Josh N. Vo[1,2,5], Steven Kregel[1,2], Stephanie A. Simko[1,2], Andrew D. Delekta[1,2], Mustapha Jaber[1], Heng Zheng[1,2], Ingrid J. Apel[1,2], Lisa McMurry[1,2], Fengyun Su[1,2], Rui Wang[1,2], Sylvia Zelenka-Wang[1,2], Sanjita Sasmal[6], Leena Khare[6], Subhendu Mukherjee[6], Chandrasekhar Abbineni[6], Kiran Aithal[6], Mital S. Bhakta[7], Jay Ghurye[7], Xuhong Cao[1,2,8], Nora M. Navone[9], Alexey I. Nesvizhskii[1,2,4,5], Rohit Mehra[1,2,4], Ulka Vaishampayan[10], Marco Blanchette[7], Yuzhuo Wang[11,12], Susanta Samajdar[6], Murali Ramachandra[6], Arul M. Chinnaiyan[1,2,4,5,8,13,*].

[1] Michigan Center for Translational Pathology, University of Michigan, Ann Arbor, MI 48109, USA.
[2] Department of Pathology, University of Michigan, Ann Arbor, MI 48109, USA.
[3] Molecular and Cellular Pathology Program, University of Michigan, Ann Arbor, MI 48109, USA.
[4] Rogel Cancer Center, University of Michigan, Ann Arbor, MI 48109, USA.
[5] Department of Computational Medicine and Bioinformatics, University of Michigan, Ann Arbor, MI 48109, USA.
[6] Aurigene Discovery Technologies Ltd., Electronic City Phase II, Bangalore 560 100, India.
[7] Dovetail Genomics, Scotts Valley, CA 95066, USA.
[8] Howard Hughes Medical Institute, University of Michigan, Ann Arbor, MI 48109, USA.
[9] Department of Genitourinary Medical Oncology and the David H. Koch Center for Applied Research of Genitourinary Cancers, The University of Texas MD Anderson Cancer Center, Houston, TX 77030, USA.
[10] Department of Internal Medicine/Oncology, University of Michigan, Ann Arbor, MI 48109, USA.
[11] Vancouver Prostate Centre, Vancouver, BC V6H 3Z6, Canada.
[12] Department of Urologic Sciences, University of British Columbia, Vancouver, BC, V6T 1Z3, Canada.
[13] Department of Urology, University of Michigan, Ann Arbor, MI 48109, USA.
[*] Correspondence to: arul@umich.edu
[†] These authors contributed equally to this work.

TABLE OF CONTENTS:

Supplementary Text……………………………………………………………...page 3-14

AU-15330 Synthesis, Chemistry, and Structure

Supplementary text and structures describing the derivation and optimization of AU-15330.

Preclinical AU-15330 Safety Evaluation in Male CD-1 Mice

Supplementary text and tables describing experiments that analyzed the safety of AU-15330 in mice.

References

Supplementary Figure 1…………………………………………………………page 15-21
Uncropped western blots from all figures.

Supplementary Table 1……………………………………………………separate Excel file
Antiproliferative half-maximal inhibitory concentrations of AU-15330 across cancer cell lines.

Supplementary Table 2……………………………………………………separate Excel file
Antibodies, qPCR primers, CRISPR/Cas9 single guide RNA sequences, short hairpin RNA sequences, and compounds used in this study.

Supplementary Table 3……………………………………………………separate Excel file
Effects of AU-15330 on hERG potassium current. Raw measurement values from three independent experiments are included. E-4031 is an experimental class III antiarrhythmic drug that blocks hERG-type potassium channels and was used as a positive control. N, number of cells tested; SEM, standard error of the mean; SD, standard deviation; +1 = whitish discoloration (slight) along the flow of the stock solution as it disperses in the external buffer and it becomes clear without any visible particles upon shaking the contents.

Supplementary Text: AU-15330 Synthesis, Chemistry, and Structure

General information: All chemicals and solvents were obtained from commercial suppliers and used without further purification. Purification was performed using combi-flash Nextgen300. All reactions were monitored by TLC, using silica gel plates with fluorescence F254 and UV light visualization. ^{1}H NMR spectra was recorded on a Varian Mercury Plus at 400 MHz, and ^{13}C NMR spectra was recorded on a JEOL-ECZ-400S spectrometer at 100 MHz. Coupling constants (J) are expressed in hertz (Hz). Chemical shifts (δ) of NMR are reported in parts per million (ppm) units relative to internal control (TMS). Signal splitting patterns are described as singlet (s), doublet (d), triplet (t), quartet (q), multiplet (m), broad (br), or a combination thereof. The low resolution of ESI-MS was recorded on an Agilent-6120 and the high-resolution mass (resolution-70000) for compound was generated using Q-Exactive Plus orbitrap system, Thermo Scientific, US using electrospray ionization (ESI). Melting point was recorded in Stuart instrument, model smp30. Specific optical rotation was recorded in an Anton Paar MCP 5100 instrument. HPLC was recorded with a Waters 2696, and the column used was a YMC Triart C-18 EXRS (150*4.6) mm 5µm using 0.01M ammonium acetate in (Aq); Mobile phase-B: ACN 100%; Method -T/%B: 0/10, 2/10, 5/85, 13/85, 14/10, 15/10 method and flow rate: 1.0 ml/min. FT-IR was recorded using PerkinElmer Spectrum.

Abbreviations used: DMSO for dimethylsulfoxide, DIPEA for *N*, *N*-diisopropylethylamine, MeOH for methanol, DMF for *N*, *N*-dimethylformamide, HATU for 1- [bis(dimethylamino) methylene]-1*H*-1,2,3-triazolo[4,5-*b*]pyridinium 3-oxid hexafluorophosphate, DCM for dichloromethane, Pd(dppf)Cl_2 for [1,1′-bis(diphenylphosphino)ferrocene]dichloro-palladium(II).

Synthesis of (2S,4R)-1-((S)-2-(2-(4-(3-amino-6-(2-hydroxyphenyl)pyridazin-4-yl)piper-azin-1-yl)acetamido)-3,3-dimethylbutanoyl)-4-hydroxy-N-((S)-1-(4-(4-methylthiazol-5-yl)phenyl)ethyl)pyrrolidine-2-carboxamide (AU-15330)

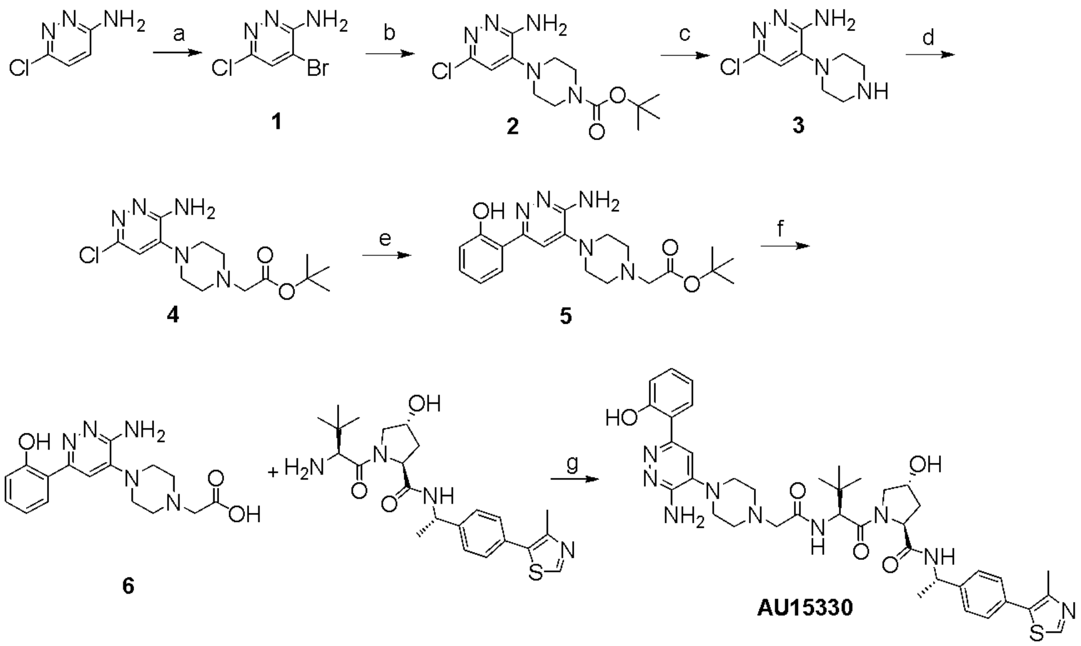

Reagents and conditions: a) Br_2, $NaHCO_3$, MeOH, 0 °C - RT, 16 h; b) DMF, 90 °C, 16h; c) 4M HCl in 1,4-dioxane, DCM, 0 °C - RT, 16h; d) DIPEA, DMF, 60 °C, 16h; e) Pd(dppf)Cl_2•DCM, K_2CO_3, dioxane, water, sealed tube, 120 °C, 6 h; f) 4 M HCl in 1,4-dioxane,

DCM, 0 °C - RT, 16h; g) HATU, DIPEA, DMF, RT, 4 h.

4-bromo-6-chloropyridazin-3-amine (1)

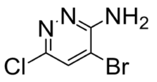

To a stirred solution of 6-chloropyridazin-3-amine (20.0 g, 155.02 mmol) in MeOH (100 mL) was added $NaHCO_3$ (19.53 g, 232.00 mmol) at RT and stirred for 15 min, and then bromine (8.74 mL, 170.52 mmol) was added dropwise to the reaction mixture over a period of 1h at 0 °C and stirred for 16h at RT. After completion of the reaction (monitored by TLC), the reaction mixture was quenched with water (100 mL). The dark-brown colored solid was precipitated, filtered, washed with water (50 mL), and dried under vacuum to afford crude **1**. The solid was washed with 20% EtOAc in hexane and diethyl ether to afford pure **1** (15.0 g, 46% yield).

^{1}H NMR (400 MHz, DMSO-*d*$_6$) δ 7.99 (s, 1H), 6.97 (bs, 2H).

MS (ESI) for $C_4H_4BrClN_3$ $[M+H]^+$ calculated 207.9, obtained 208.0.

tert-butyl 4-(3-amino-6-chloropyridazin-4-yl)piperazine-1-carboxylate (2)

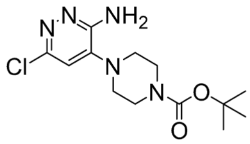

To a stirred solution of 4-bromo-6-chloropyridazin-3-amine **1** (20.0 g, 96.66 mmol) in DMF (400 mL) was added tert-butyl piperazine-1-carboxylate (53.92 g, 289.9 mmol) at RT and stirred for 16h at 90 ºC under nitrogen atmosphere. Then the reaction mixture was quenched with cold water (200 mL), and the brown solid obtained was washed with diethyl ether, filtered, and dried under vacuum. The same procedure was repeated four times to afford pure **2** (15.0 g, 49.57% yield).

^{1}H NMR (400 MHz, DMSO-*d*$_6$) δ 6.91 (s, 1H), 6.21 (s, 2H), 3.49 (t, J = 4.4 Hz, 4H), 2.92 (t, J = 4.8 Hz, 4H), 1.41 (m, 9H).

MS (ESI) for $C_{13}H_{21}ClN_5O_2$ $[M+H]^+$ calculated 314.1, obtained 314.2.

6-chloro-4-(piperazin-1-yl)pyridazin-3-amine hydrochloride (3)

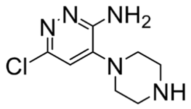

To a stirred solution of tert-butyl 4-(3-amino-6-chloropyridazin-4-yl)piperazine-1-carboxylate **2** (15.0 g, 47.92 mmol) in DCM (100 mL) was added 4 M HCl in 1,4-dioxane (75 mL) at 0 °C under nitrogen atmosphere and stirred for 16h at RT. The reaction mixture was concentrated

under reduced pressure to get crude brown solid compound. The brown solid was washed with diethyl ether (2x 50 mL), filtered, and dried under vacuum to afford **3** (11.98 g, 100% yield).

^{1}H NMR (400 MHz, DMSO-*d$_6$*) δ 9.44 (bs, 2H), 7.75 (bs, 2H), 7.32 (s, 1H), 3.30-3.25 (m, 8H).

MS (ESI) for $C_8H_{13}ClN_5$ $[M+H]^+$ calculated 214.1, obtained 214.1.

tert-butyl 2-(4-(3-amino-6-chloropyridazin-4-yl)piperazin-1-yl)acetate (4)

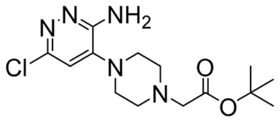

To a stirred solution of 6-chloro-4-(piperazin-1-yl)pyridazin-3-amine hydrochloride **3** (12.0 g, 48.18 mmol) in DMF (100 mL) in a sealed tube were added DIPEA (25.70 mL, 144.54 mmol) and tert-butyl 2-bromoacetate (10.53 mL, 72.27 mmol) at RT and stirred for 16h at 60 ºC. The reaction mixture was quenched with water (100 mL) and extracted with EtOAc (2 X 150 mL). The combined organic layer was washed with water (100 mL), brine (100 mL), dried over anhydrous sodium sulphate, and concentrated under reduced pressure to give the crude product which was purified by combi flash using 60% ethyl acetate in hexane as eluent to afford **4** (10.0 g, 63.7% yield).

^{1}H NMR (400 MHz, DMSO-*d$_6$*) *δ* 6.89 (s, 1H), 6.07 (s, 2H), 3.165 (s, 2H), 3.05-2.95 (m, 4H) 2.70-2.65 (m, 4H), 1.45 (s, 9H).

MS (ESI) for $C_{14}H_{23}ClN_5O_2$ $[M+H]^+$ calculated 328.2, obtained 328.2.

tert-butyl 2-(4-(3-amino-6-(2-hydroxyphenyl)pyridazin-4-yl)piperazin-1-yl)acetate (5)

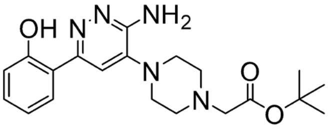

To a stirred solution of tert-butyl 2-(4-(3-amino-6-chloropyridazin-4-yl)piperazin-1-yl)acetate **4** (1.8g, 5.47 mmol) and (2-hydroxyphenyl)boronic acid (1.7g, 10.94 mmol) in 1,4-dioxane (20 mL) was added K_2CO_3 (2M) solution (4.52 g, 32.8 mmol) and degassed with nitrogen for 5 min. Pd(dppf)Cl_2•DCM (0.44 g, 0.54 mmol) was added, and the reaction mixture was heated for 6h at 120 ºC in a sealed tube. Once the reaction was completed (monitored by TLC), the reaction mixture was diluted with EtOAc. The combined organic layer was washed with water, brine, dried over anhydrous sodium sulphate, and concentrated under vacuum to give the residue which was purified by combi flash column chromatography using 50-60% ethyl acetate in hexane as eluent to afford **5** (0.9 g, 63.7% yield).

^{1}H NMR (400 MHz, DMSO-*d$_6$*) *δ* 14.15 (s, 1H), 7.94 (dd, J_1 = 1.6, J_2 = 8.4 Hz,1H), 7.26-7.21 (m, 2H), 6.90-6.87 (m, 2H), 6.22 (s, 2H), 3.18-3.16 (m, 2H), 3.11-3.09 (m, 4H), 2.67-2.66 (m, 4H), 1.43 (s, 9H).

MS (ESI) for $C_{20}H_{28}N_5O_3$ $[M+H]^+$ calculated 386.2, obtained 386.1.

2-(4-(3-amino-6-(2-hydroxyphenyl)pyridazin-4-yl)piperazin-1-yl)acetic acid (6)

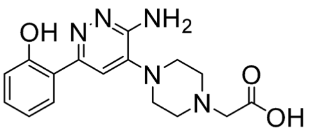

To a stirred solution of tert-butyl 2-(4-(3-amino-6-(2-hydroxyphenyl)pyridazin-4-yl)piperazin-1-yl)acetate **5** (5 g, 0.258 mmol.) in DCM (50 vol.) was added 4 M HCl in 1, 4-Dioxane (75 mL) at 0 °C under nitrogen atmosphere and stirred for 16h at RT. After completion of the reaction (monitored by TLC), the reaction mixture was concentrated under reduced pressure. The residue was washed with diethyl ether, filtered, and dried under vacuum to afford compound **6** (4.27 g, 95% yield).

^{1}H NMR (400 MHz, DMSO-d_6) δ 7.72 (s, 1H), 7.64 (d, J_1 = 1.6, J = 7.6 Hz, 1H), 7.43 (t, J = 7.6 Hz, 1H), 7.04 (m, 2H), 4.25 (s, 2H), 3.72 (bs, 8H).

MS (ESI) for $C_{16}H_{20}N_5O_3$ $[M+H]^+$ calculated 330.2, obtained 330.1.

(2S,4R)-1-((S)-2-(2-(4-(3-amino-6-(2-hydroxyphenyl)pyridazin-4-yl)piperazin-1-yl)acetamido)-3,3-dimethylbutanoyl)-4-hydroxy-N-((S)-1-(4-(4-methylthiazol-5-yl)phenyl)ethyl)pyrrolidine-2-carboxamide (AU-15330)

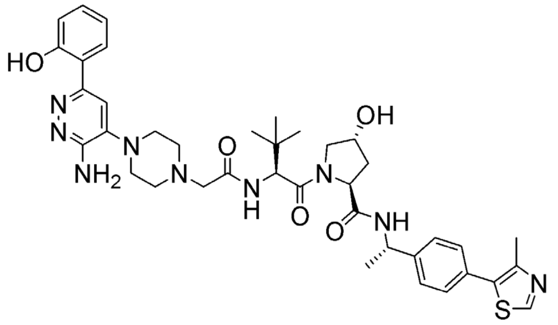

To a stirred solution of 2-(4-(3-amino-6-(2-hydroxyphenyl)pyridazin-4-yl)piperazin-1-yl)acetic acid **6** (0.3 g, 0.91 mmol) and (2S,4R)-1-((S)-2-amino-3,3-dimethylbutanoyl) -4-hydroxy-N-((S)-1-(4-(4-methylthiazol-5-yl)phenyl)ethyl)pyrrolidine-2-carboxamide (0.65 g, 1.36 mmol) in DMF (3 mL) was added HATU (0.51 g, 1.336 mmol) at 0 ºC followed by the dropwise addition of DIPEA (0.65 mL, 3.64 mmol). Stirring was continued at RT for 16h. After completion of the reaction (monitored by TLC), the reaction mixture was poured into crushed ice. The solid formed was filtered off to get the crude product which was purified by combi flash using 4 % MeOH in DCM as eluent to afford **AU-15330** as a pale yellow solid (0.25 g, 37% yield).

m.p.: 156-159 ºC; TLC ($CHCl_3$: MeOH, 90:10 v/v): RF = 0.45; $[\alpha]^D{}_{20}$ (deg cm^3 g^{-1} dm^{-1}) = -49.118 ° (c = 0.1018 g /100 cm^3 in ethanol); HPLC purity 99.4% (RT- 6.54 min).

^{1}H NMR (400 MHz, DMSO-d_6) δ 14.22 (s, 1H), 8.97 (s, 1H), 8.41 (d, J = 7.8 Hz, 1H), 7.95 (d,

J = 7.4 Hz, 1H), 7.77 (d, J = 9.8 Hz, 1H), 7.56 (s, 1H), 7.43 (d, J = 8.3 Hz, 2H), 7.36 (d, J = 8.3 Hz, 2H), 7.25 (t, J = 6.8 Hz, 1H), 6.88 (dd, J_1 = 5.8 Hz, J_2 = 4.4 Hz, 2H), 6.25 (s, 2H), 5.11 (d, J = 3.5 Hz, 1H), 4.89 (d, J = 7.3 Hz, 1H), 4.53 (d, J = 9.8 Hz, 1H), 4.44 (t, J = 7.8 Hz, 1H), 4.29 (bs, 1H), 3.60 (d, J = 5.9 Hz, 2H), 3.17 (bs, 4H), 3.13 (s, 1H), 3.0 (d, J = 16.0 Hz, 1H), 2.69 (dd, J_1 = 4.4 Hz, J_2 = 2.0 Hz, 4H), 2.45 (s, 3H), 2.08 (d, J = 13.2 Hz, 1H), 1.81-1.74 (m, 1H), 1.36 (d, J = 6.9 Hz, 3H), 0.96 (s, 9H).

^{13}C NMR (100 MHz, DMSO-d_6) δ 170.4, 169.1, 168.4, 158.5, 154.6, 153.2, 151.5, 147.7, 144.7, 140.1, 131.1, 130.1, 129.6, 128.8, 126.3, 118.4, 117.7, 117.3, 110.6, 68.7, 60.7, 58.5, 56.5, 55.8, 52.4, 48.5, 47.7, 40.1, 39.9, 39.0, 38.8, 37.7, 35.8, 26.3, 22.5, 15.9.

IR (KBr): 3356.88, 2959.95, 1627.5, 1575.95, 1523.03, 1425.09, 1192.61, 1014.27, 970.79, 836.52, 755.45 cm^{-1}.

HRMS (ESI) for $C_{39}H_{49}N_9O_5S$ $[M+H]^+$ calculated 756.3611, found 756.3635.

NMR spectra of AU-15330

S1: ^{1}H NMR of (AU-15330)

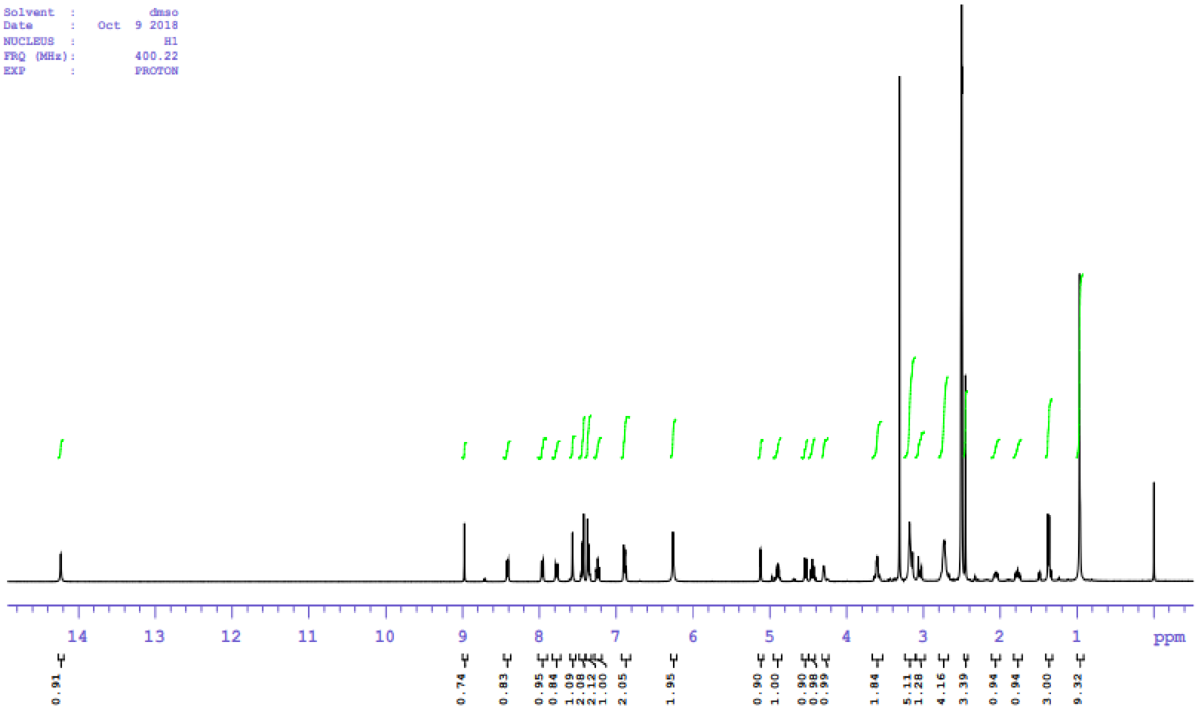

S2: ^{13}C NMR of (AU-15330)

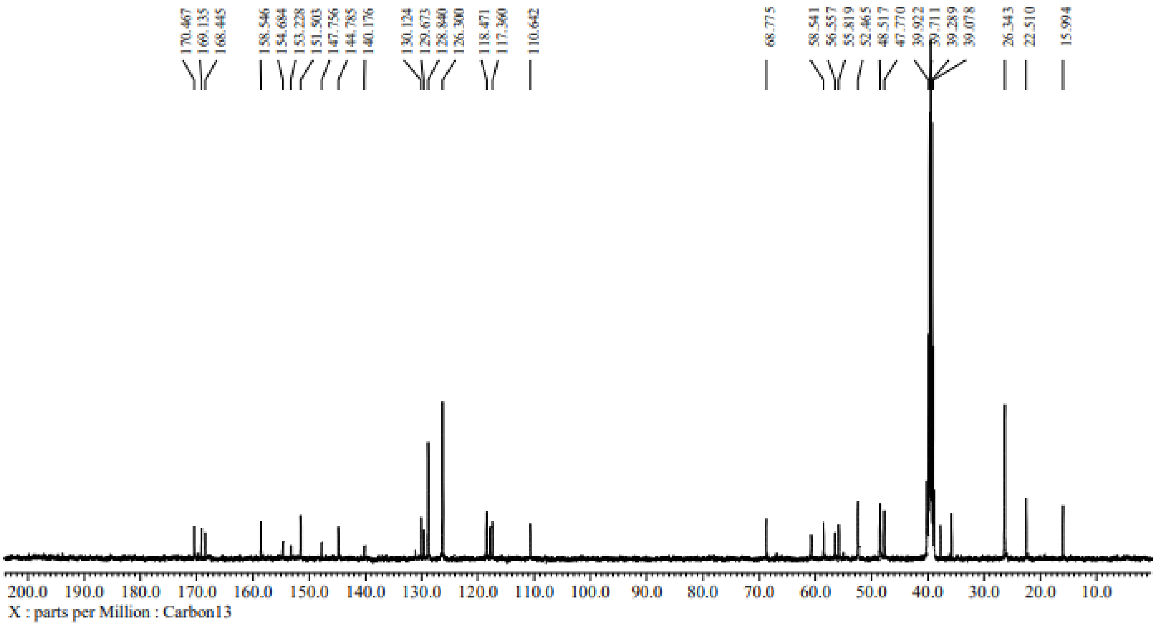

S3: HPLC of (AU-15330)

HPLC Method conditions:
Column: YMC Triart C-18 EXRS (150*4.6)mm 5µm
Mobile phase-A:0.01M Ammonium acetate in (Aq); Mobile phase-B:ACN 100%
Method -T/%B: 0/10, 2/10, 5/85, 13/85, 14/10, 15/10
Flow rate: 1.0 ml/min
Column temp: 30°C
Diluent: ACN+H2O

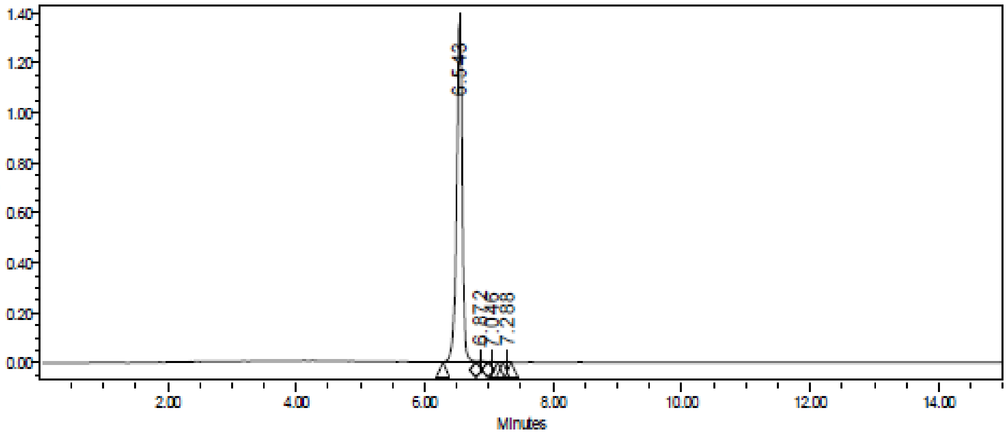

Peak Results

	RT	Area	% Area
1	6.543	8285835	99.40
2	6.872	33920	0.41
3	7.046	7249	0.09
4	7.288	8604	0.10

S4: FT-IR of (AU-15330)

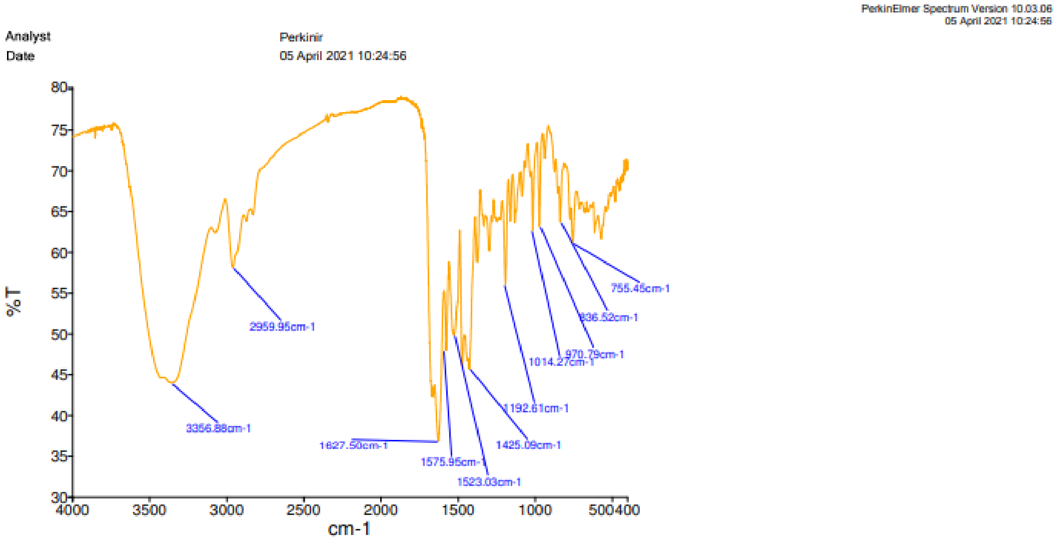

Molecular modeling strategy for designing AU-15330

The binding model of AU-15330 in complex with SMARCA2-BD and VHL was generated using Aurigene's proprietary computing algorithm, ALMOND (ALgorithm for MOdeling Neosubstrate Degraders) (DOI: 10.31031/MADD.2021.03.000560). The algorithm was developed using the ICM-Pro integrated modeling platform (http://www.molsoft.com/icm_pro.html) and trained to predict models of ternary complexes of bi-functional molecules with very short or no linkers. The process employs protein-protein docking simulation, exhaustive conformational sampling, small molecule-protein docking, and site-directed scoring of predicted ternary complex models.

MF Score (mean force score) provides an independent score of the strength of ligand-receptor interaction, which signifies the strength of the induced protein-protein interaction in the target – E3 ligase complex, and a lower score suggests a stronger binding. A cut off score of less than -250 using this algorithm was considered for prioritization. Validation of this hypothesis has been exemplified in the following table.

Compound Code	Structure	MF Score	%Degradation of SMARCA2 at 10 nM (H838 cells)
**AU-15330**	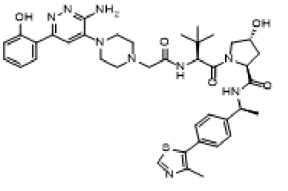	**-291.1**	**90%**
**Compound 2**	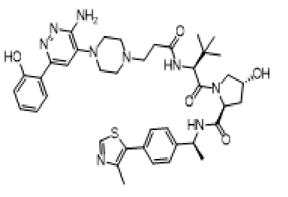	**-278.4**	**22%**
**Compound 3**	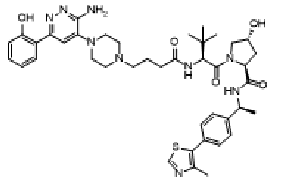	**-221.6**	**No degradation**

Supplementary Text: Preclinical AU-15330 Safety Evaluation in Male CD-1 Mice

Vehicle and formulation

Dose (0, 10, & 30 mg/kg/day)	10% w/v HPβCD + 5% w/v dextrose + purified water q.s.
Dose (60 mg/kg, (2+/5-) for 2 cycles or once every three days)	10% w/v HPβCD + 5% w/v dextrose + purified water q.s.<br>(pH adjusted to ~4.5 to 4.8 with 1N HCl)

Intravenous formulation preparation details

The test item AU-15330 was formulated in 10% w/v HPβCD + 5% w/v dextrose + purified water q.s for 10 and 30 mg/kg doses. AU-15330 was formulated in 10% w/v HPβCD + 5% w/v dextrose + purified water q.s with pH adjusted to ~4.5 to 4.8 with 1N HCl for 60 mg/kg infrequent intravenous administration. Formulation was stored at room temperature (~25°C) in a screw-capped glass bottle until use and dosed within 2h of its preparation.

Test system

Male Hsd: ICR (CD-1®) mouse of 7-9 weeks age (6 animals/group, main study and 3 animals/group, toxicokinetic satellite group) were selected as test species.

Treatment

AU15330 was administered once daily up to 7 days at doses of 0, 10, and 30 mg/kg/day by slow (10 sec to 15 sec) intravenous injection through the tail vein. In the intermittent dosing study, the frequency of dose administration, duration of treatment, and dosing procedure was followed as shown below in the figure (2 on/5 off and once every three days dosing for 2 cycles at 0, 60 mg/kg by slow (10 sec to 15 sec) intravenous injection through the tail vein). The dose volume was 5 mL/kg for all groups.

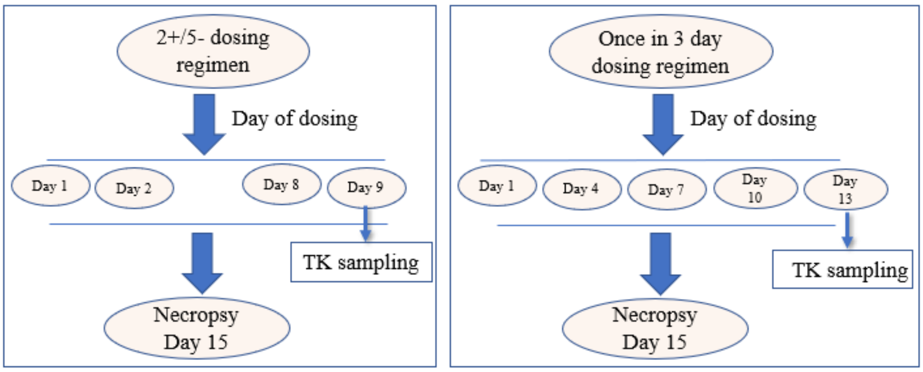

Mortality/moribundity

Animals were observed for mortality/moribundity at least once daily during the acclimatization period and twice daily during the dosing period (once in the morning hours and once in the afternoon hours).

Clinical signs of toxicity

A routine clinical examination was performed at least once daily during the acclimatization period and twice daily during the dosing period (once in the morning hours (~ within 30 mins of dosing) and once in the afternoon hours).

Body weight
Individual animal body weights were recorded daily using a weighing balance connected to a printer throughout the pretest treatment periods.

Food consumption
Pre-weighed amounts of 50 g of food was offered to each cage daily, and the quantity of food left in each cage was weighed approximately after 24 hours each day throughout the experimental period. Food consumption (g/mice/day) was calculated as below:

$$\text{Food consumption (g/mice/day)} = \frac{\text{Total food offered (g)} - \text{Food left over (g)}}{\text{No. of surviving animals}}$$

Clinical pathology (blood sampling procedure)
At scheduled necropsy, mice were anesthetized by isoflurane. Blood samples were collected from the inferior vena cava and animals were exsanguinated after the opening of the abdominal vessels. Food was withdrawn 4-6 hours before blood collection.

Group-wise animals were anesthetized in sequence with isoflurane ~3-5 mins before blood collection, and blood samples were collected from the inferior vena cava after the opening of the abdominal cavity. Blood samples from each animal were collected into two different tubes, one for hematology and another for clinical chemistry. Additionally, blood smears were prepared for further evaluation from all groups.

Hematology analysis
For evaluation of hematology parameters, approximately 0.4 mL of blood was collected into tubes containing ethylenediaminetetraacetic acid dipotassium salt dihydrate solution as an anticoagulant (2% K2 EDTA, pH 7.4). The following parameters were measured using ADVIA 2120 hematology analyzer (Siemens):

**Parameter**	**Unit**
Hemoglobin (HGB)	g/dL
Red blood cell counts (RBC)	x 10^6 cells/µL
Hematocrit (HCT)	%
Mean corpuscular hemoglobin (MCH)	pg
Mean corpuscular hemoglobin concentration (MCHC)	g/dL
Mean corpuscular volume (MCV)	fL
Platelet count (PLT)	x 10^3 cells/µL
Total white blood cell count (WBC)	x 10^3 cells/µL
Mean Platelet Volume (MPV)	fL
Differential white blood cell count<br>Neutrophil (N)<br>Lymphocytes (L)<br>Basophil (B)<br>Eosinophil (E)<br>Monocyte (M)	% and x 10^3 cells/µL
Reticulocytes count	% and x 10^9 cells/L

Clinical chemistry analysis

For clinical biochemistry estimations, approximately 0.6 mL of blood was collected in plain tubes and allowed to clot at room temperature for ~1 hour. The serum was separated by centrifugation at 5000 rpm for 10 minutes at about 5 °C. The following parameters were measured:

Parameter in Serum	Unit	Abbreviation	Instrument
Glucose	mg/dL	GLUC	(a)
Urea	mg/dL	UREA	(a)
Creatinine	mg/dL	CREA	(a)
Creatine Kinase	U/L	CK	(a)
Total bilirubin	mg/dL	T BIL	(a)
Cholesterol	mg/dL	CHOL	(a)
Triglycerides	mg/dL	TRIGS	(a)
Total protein	g/dL	TP	(a)
Albumin (A)	g/dL	ALB	(a)
Globulin (G)	g/dL	GLOB	Total protein-albumin
Albumin/globulin ratio	NA	A/G Ratio	albumin/globulin
Alanine aminotransferase	U/L	ALT	(a)
Aspartate aminotransferase	U/L	AST	(a)
Alkaline phosphatase	U/L	ALP	(a)
Gamma-glutamyl transferase	U/L	GGT	(a)
Lactate dehydrogenase	U/L	LDH	(a)
Aldolase	U/L	ALS	(a)

Instrument:

a) RX monaco, Randox Autoanalyzer

NA- Not Applicable

Bioanalysis and toxicokinetics (TK)

Blood sampling time points and bleeding schedule

For daily dosing study (TK sampling time point was followed as mentioned below): On day 1, TK study animals from each test item-treated dose group were bled at 0, 0.25, 1, 3, 7, and 24 hours post-dose from the saphenous vein for drug level estimations with n=3 animals per time point. On day 7, all animals from each test item-treated dose group along with control animals were bled at 0, 0.25, 1, 3, 7, and 24 hours post-dose from the saphenous vein for drug level estimations with n=3 animals per time point. Additionally, 0.08 & 0.5 hr time points were part of intravenous sampling.

For intermittent dosing study (TK sampling time point was followed as mentioned below): All animals from each test item-treated dose group along with control animals were bled at each time point on day 9 (2 on/5 off) and day 13 (for once every three days dosing) at 0, 0.08, 0.5, 1, 3, 7, and 24 hours post-dose from a saphenous vein for drug level estimations.

Approximately 0.60-0.80 mL of blood samples were collected in 2% K2EDTA (as an anticoagulant) and centrifuged at 6000 rpm for 5 minutes at 5 °C to separate the plasma. Plasma samples for drug level estimations were stored at -80 °C until analysis.

Bioanalysis and TK analysis

The analysis was carried out using the LC/MS-MS bioanalytical method (qualified fit for the purpose method). The TK parameters such as C_{max}/C_0, $AUC_{(0-t)}$, and T_{max} were determined by Non-Compartmental Analysis using Phoenix version 8.0 (Pharsight Corporation, USA).

Necropsy examination

A detailed necropsy examination was performed ~4-6 hour fasting for all treated animals. The animals were weighed just before necropsy, and this weight was used for relative organ weight calculations. Approximately 4 hours after the last dosing, blood was collected under isoflurane anesthesia for hematology in 2% K2 EDTA solution and in plain tubes to separate serum for biochemistry. After blood collection, the animals were necropsied, and a detailed necropsy examination was carried out by a veterinary pathologist. The necropsy included an examination of the external surface, external orifices, abdominal, thoracic, and cranial cavities, organs, and tissues. On completion of the gross pathology examination, the tissues were collected, weighed, and preserved from all animals.

Histopathological examination

In the daily dosing study, the following tissues were evaluated microscopically: liver, kidneys, heart, gastrocnemius muscle, stomach, testes, spleen, thymus, mesenteric lymph nodes, injection site, femur, bone marrow smear, and blood smear from all groups.

In the intermittent dosing study, the following tissues were evaluated microscopically: liver, spleen, thymus, mesenteric lymph nodes, pancreas, femur, bone marrow smear, and blood smear from all groups.

Results summary

As AU-15330 degrades mouse orthologs of its SWI/SNF targets in murine cell lines (**Extended Data Fig. 9a**), we carried out a comprehensive assessment of AU-15330 tolerability using different dosages as well as dosing patterns in immuno-competent, non-tumor bearing CD-1 mice (**Extended Data Fig. 9b**). Notably, AU-15330 was robustly detected in plasma for several hours after intravenous administration (**Extended Data Fig. 9c**) and triggered a marked loss in SMARCA2 and SMARCA4 proteins in normal mouse tissues within 5 days of treatment (**Extended Data Fig. 9d**). Yet, AU-15330 treatment over a period of 2 weeks in mice resulted in no major changes in either whole body weights (**Extended Data Fig. 9e**) or weights of essential visceral organs (**Extended Data Fig. 9f**). Furthermore, we found no reduction in the levels of white or red blood cells or blood platelets (**Extended Data Fig. 9g**), acute depletion of the latter being previously reported for the first-generation of Bromodomain and Extra-Terminal motif (BET) family inhibitors[1,2]. AU-15330 treatment also had no inhibitory effect on the functioning of the hERG-type potassium channels, mitigating the possibility of cardiac related side-effects (E-4031 was used as positive control; **Supplementary Table 3**).

References:

1. Asangani, I. A. *et al.* BET Bromodomain Inhibitors Enhance Efficacy and Disrupt Resistance to AR Antagonists in the Treatment of Prostate Cancer. *Mol. Cancer Res.* **14**, 324–331 (2016).
2. Faivre, E. J. *et al.* Selective inhibition of the BD2 bromodomain of BET proteins in prostate cancer. *Nature* **578**, 306–310 (2020).

Figure 1b

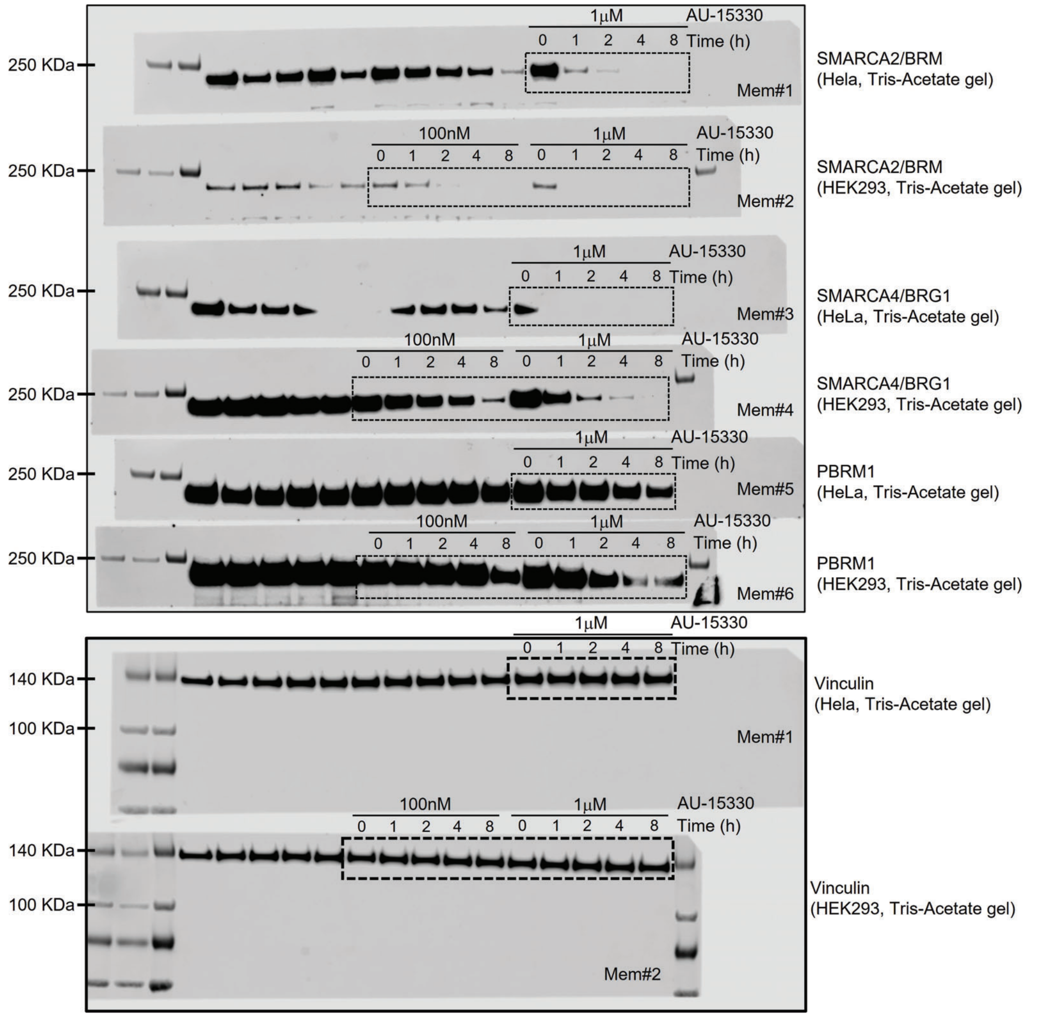

Figure 3a

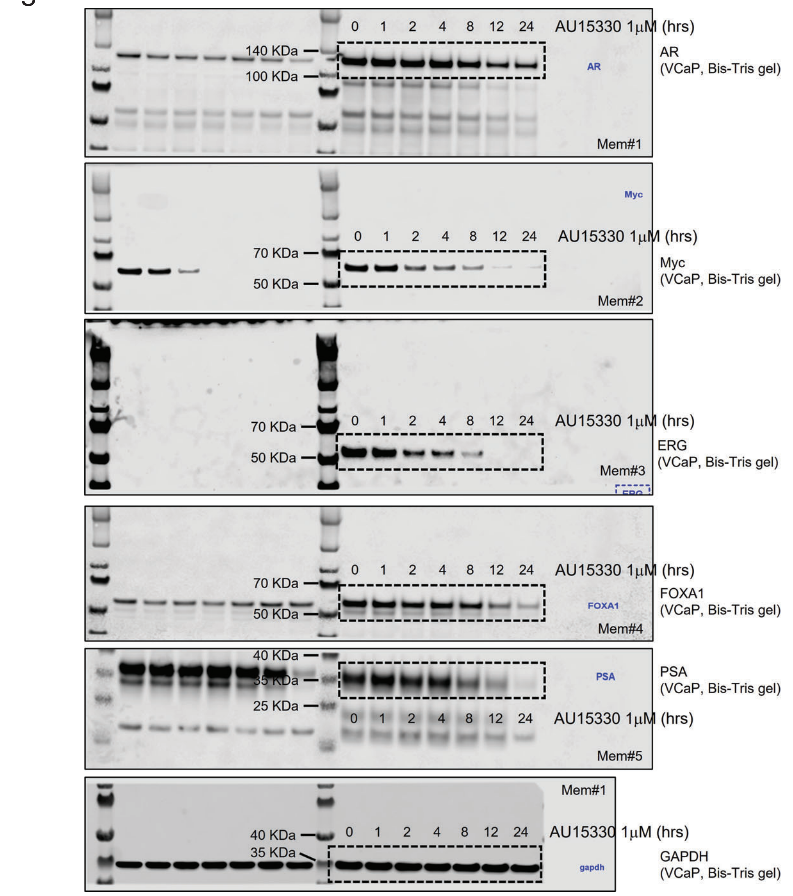

Extended data Figure 1i

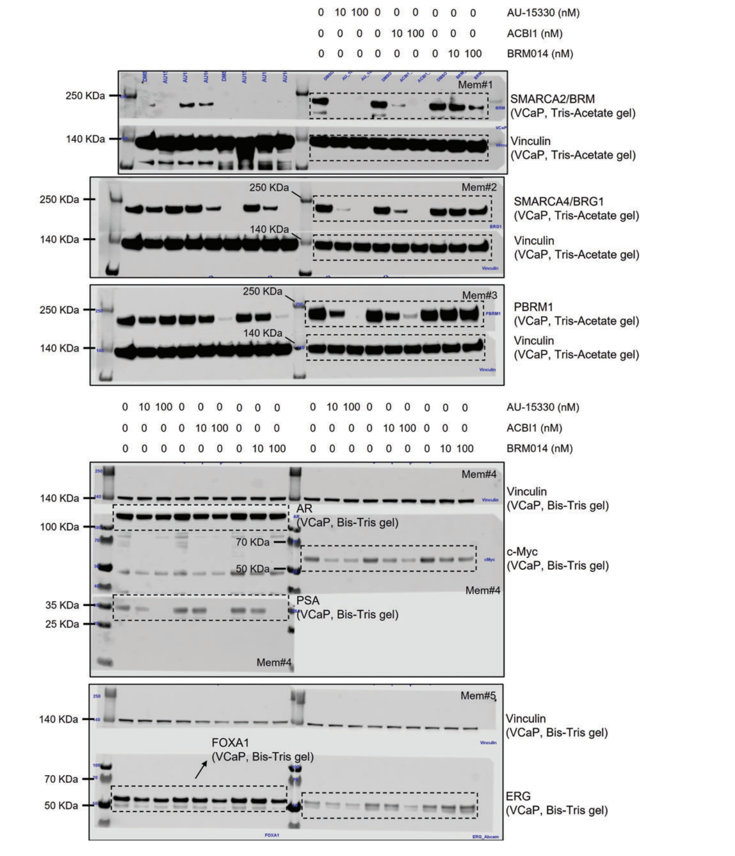

Extended data Figure 1k

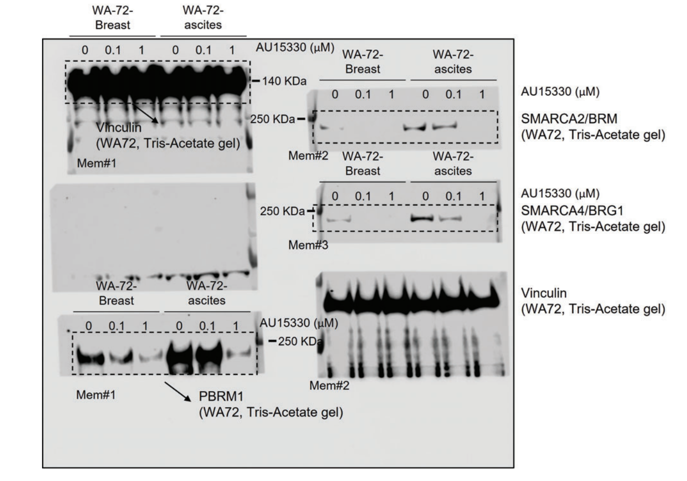

Extended data Figure 2a

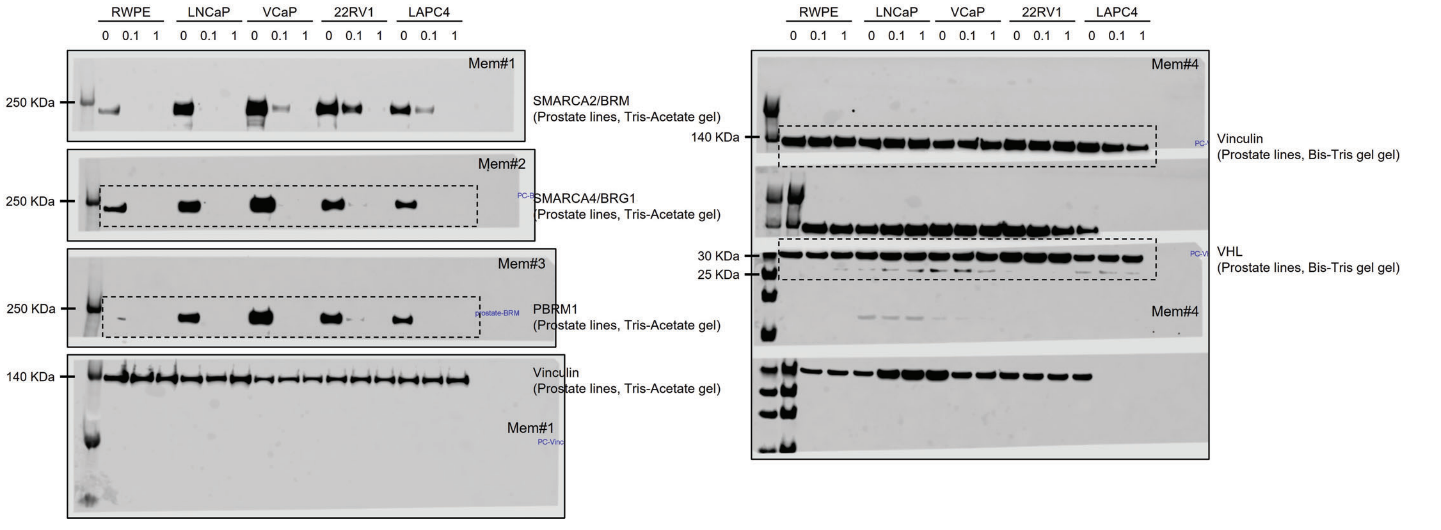

Extended data Figure 2b

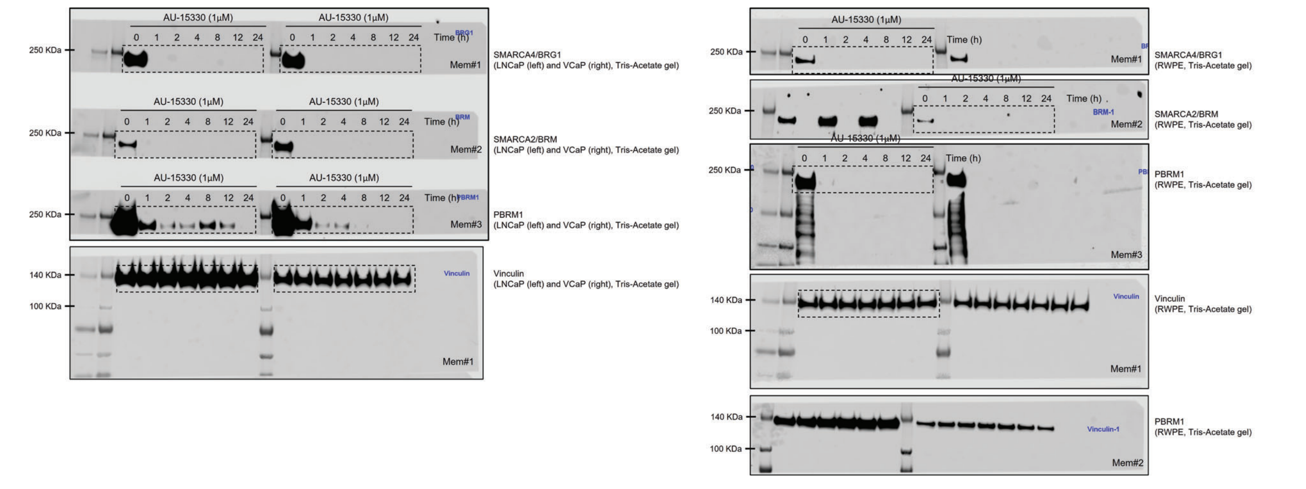

Extended data Figure 2c

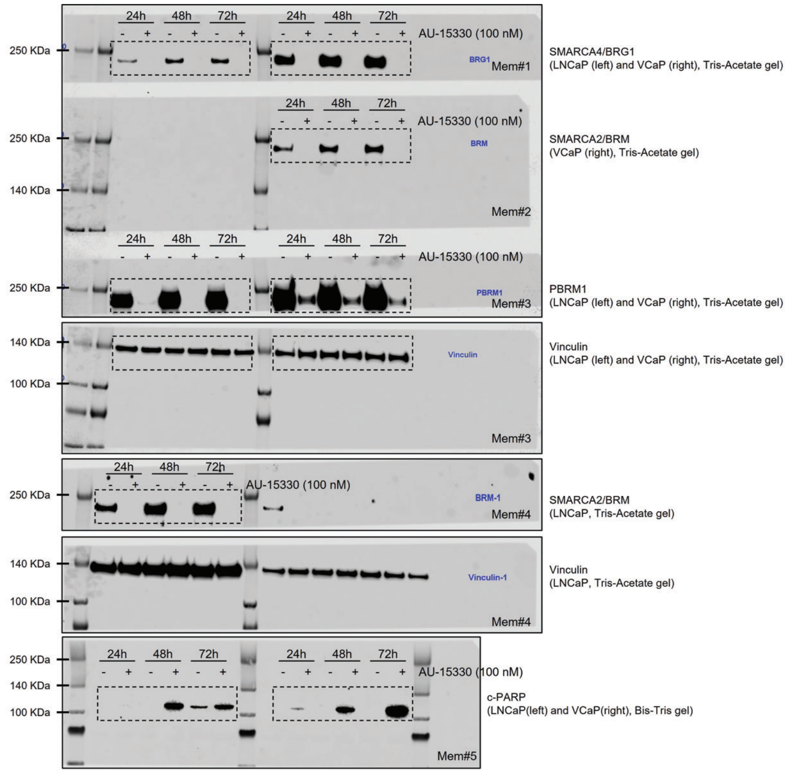

Extended data Figure 2f

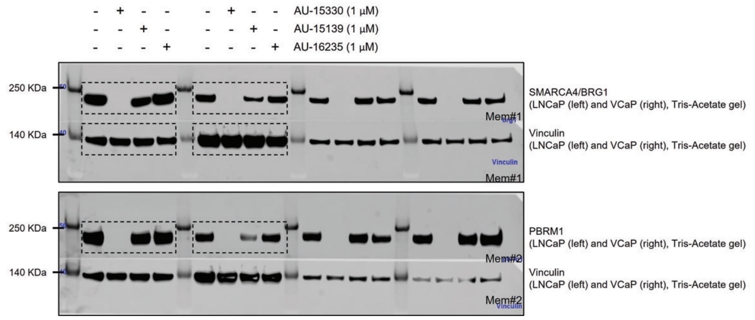

Extended data Figure 2g

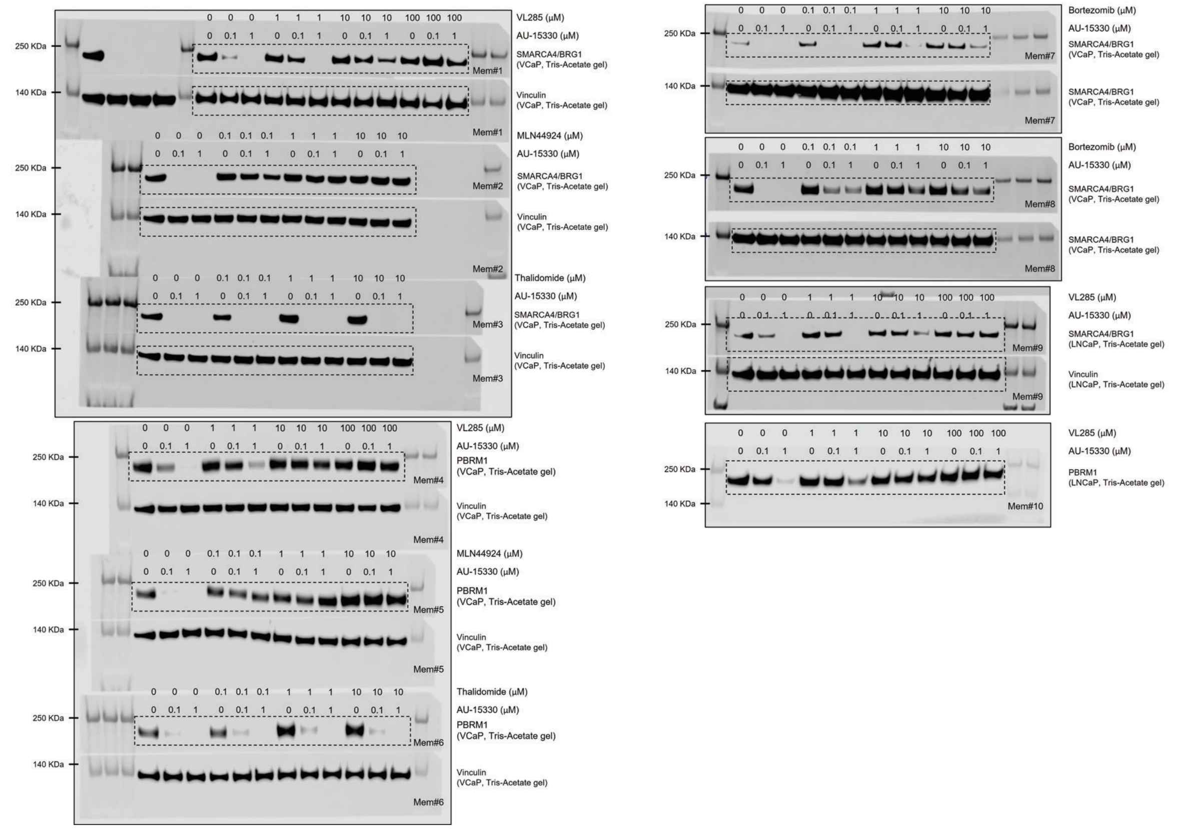

Extended data Figure 3a

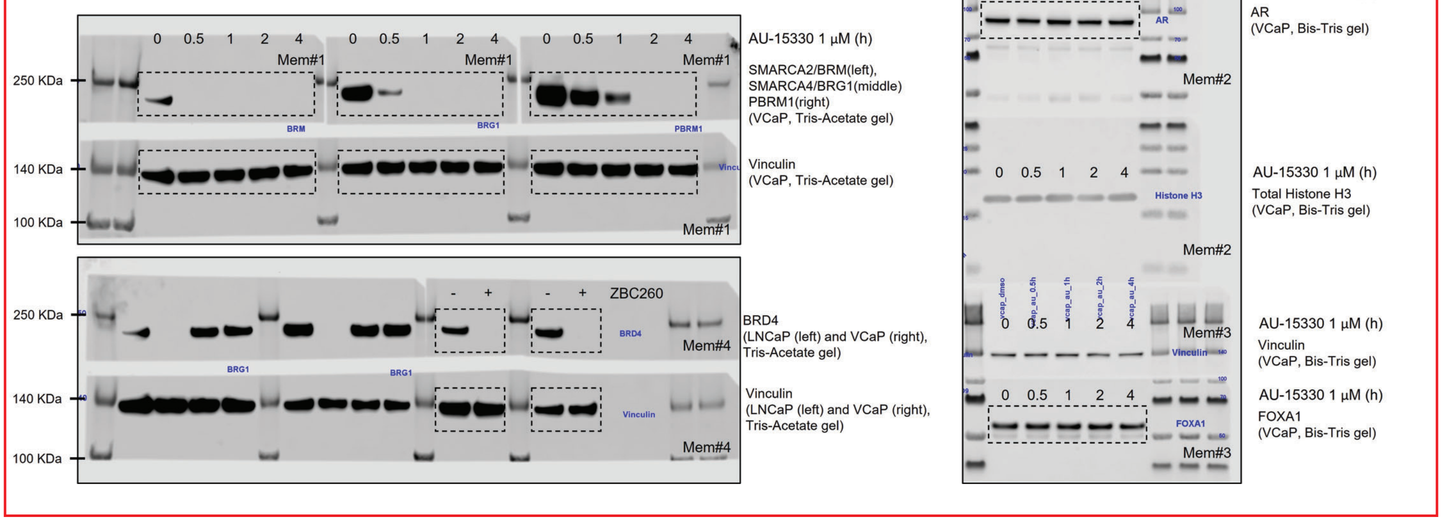

Extended data Figure 3c

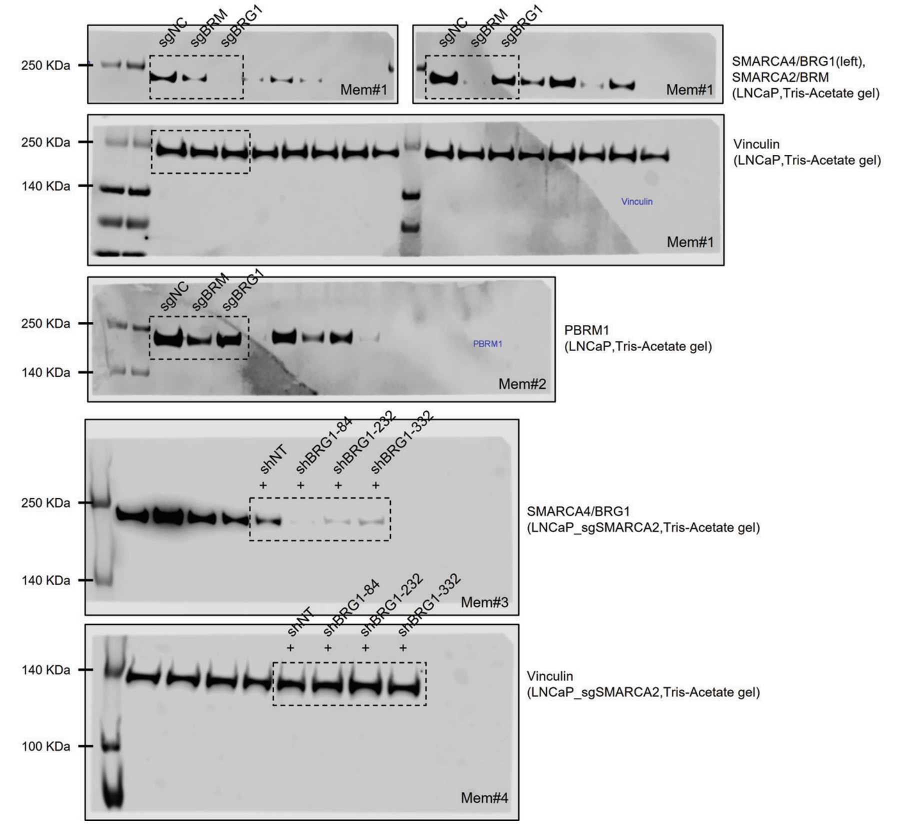

Extended data Figure 4f

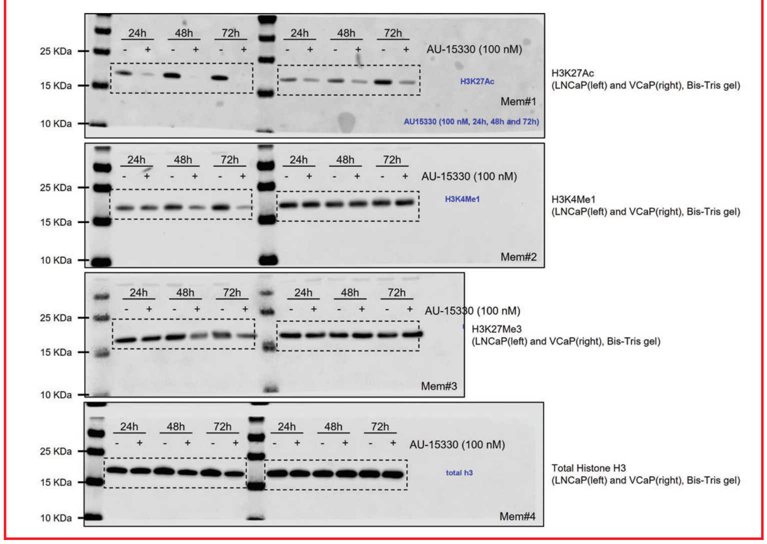

Extended data Figure 5e

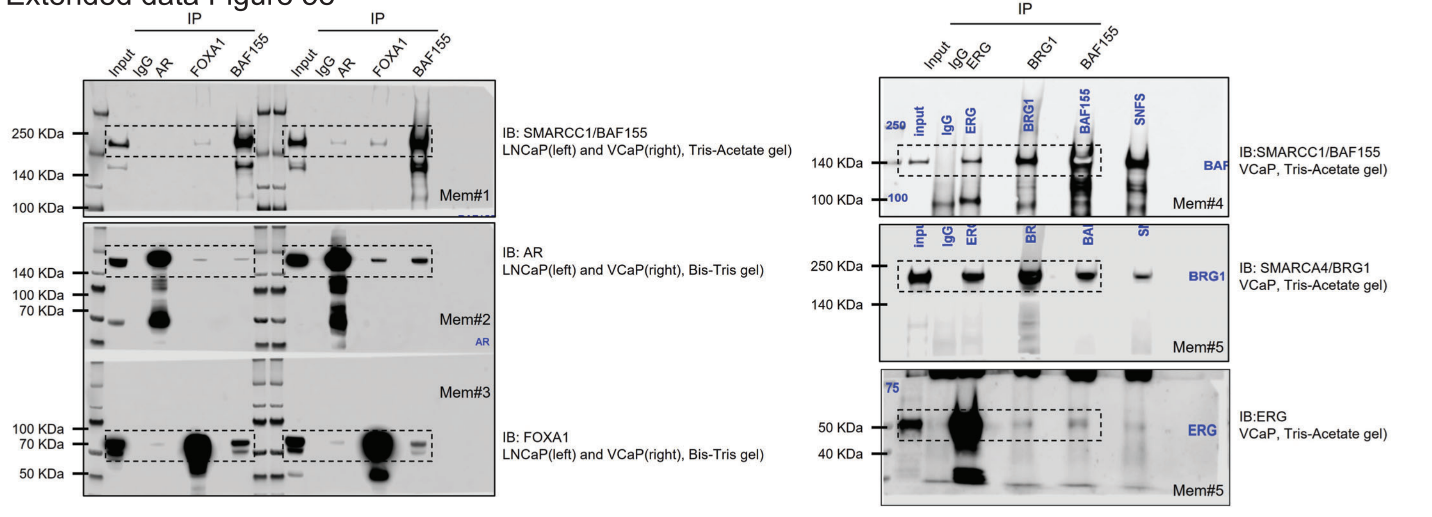

Extended data Figure 6b and 6d

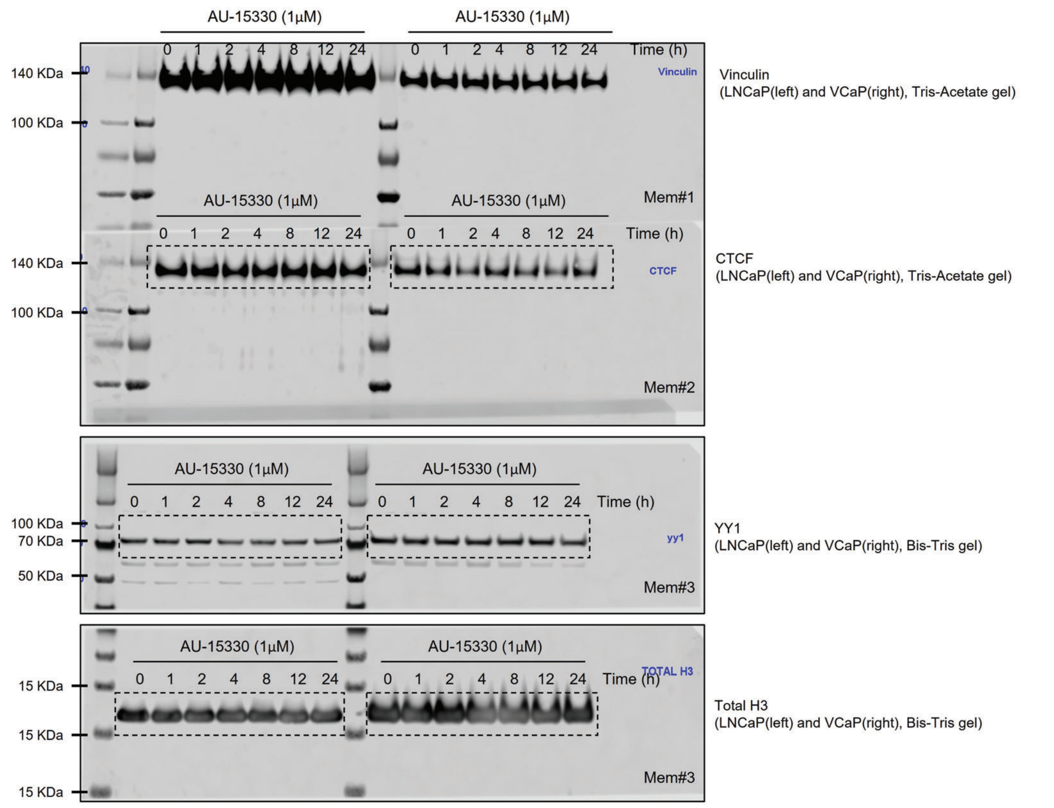

Extended data Figure 6j

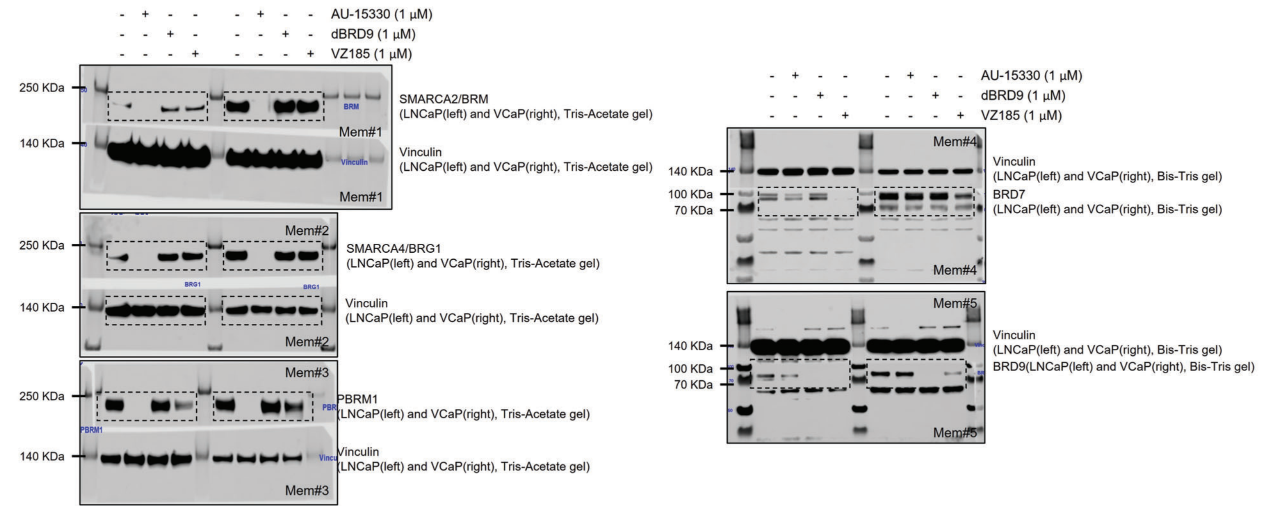

Extended data Figure 7c

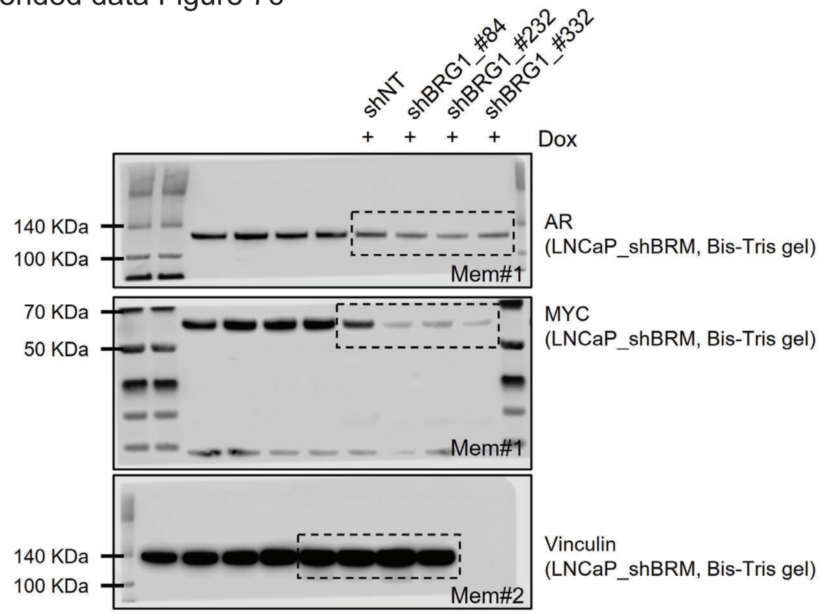

Extended data Figure 9a

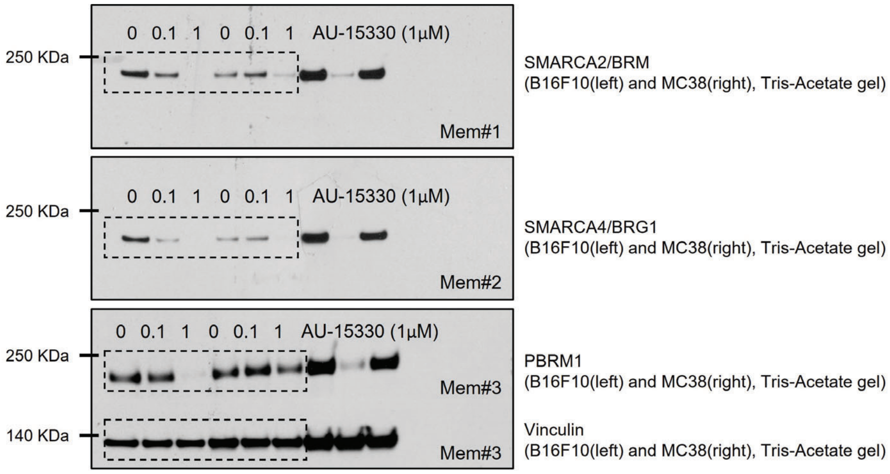

Extended data Figure 10c

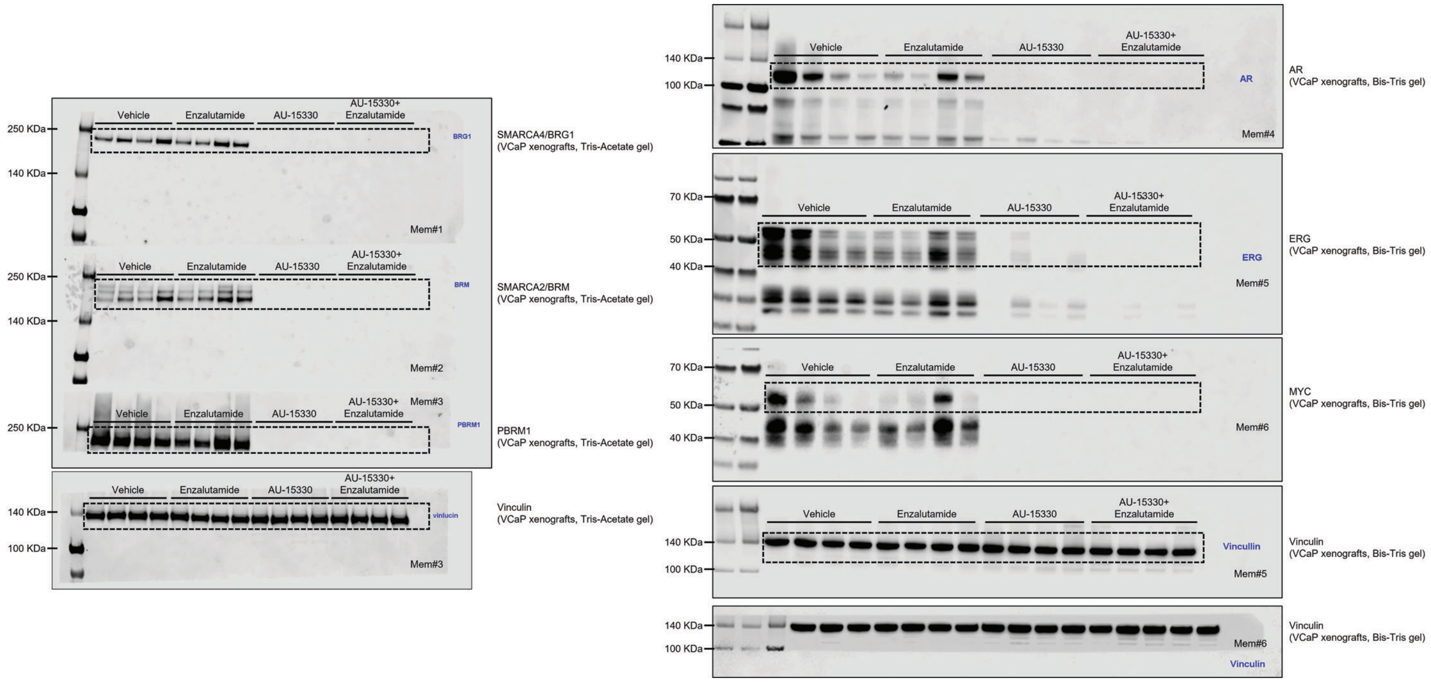

Extended data Figure 11d

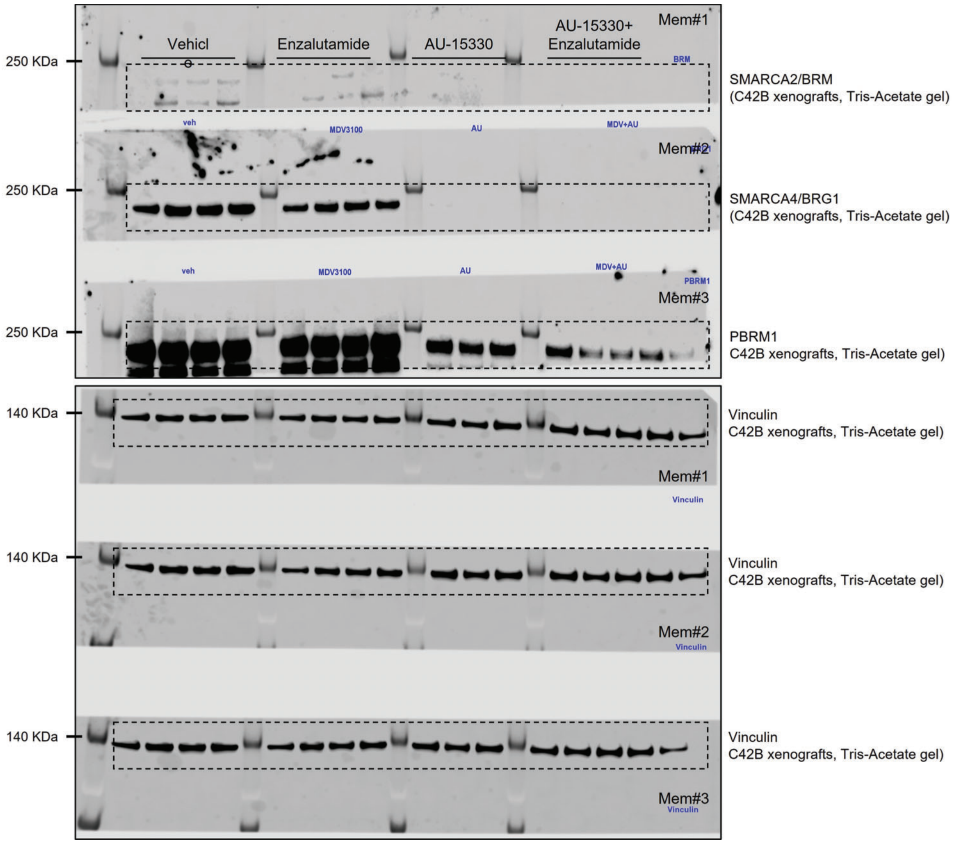

Supplement: Supplementary file 1 — This file contains Supplementary Text and Supplementary Figure 1 (uncropped western blots from all figures). [file 41586_2021_4246_MOESM1_ESM.pdf]
