# Supplementary material for: Targeting SWI/SNF ATPases in enhancer-addicted prostate cancer
Source: Nature. 2021 Dec 22;601(7893):434–9. doi: 10.1038/s41586-021-04246-z (PMC8770127; doi:10.1038/s41586-021-04246-z)
Supplement: Supplementary file 2 — Reporting Summary [file 41586_2021_4246_MOESM2_ESM.pdf]

nature research

Corresponding author(s): Arul M. Chinnaiyan

Last updated by author(s): Oct 28, 2021

Reporting Summary

Nature Research wishes to improve the reproducibility of the work that we publish. This form provides structure for consistency and transparency in reporting. For further information on Nature Research policies, see our Editorial Policies and the Editorial Policy Checklist.

Statistics

For all statistical analyses, confirm that the following items are present in the figure legend, table legend, main text, or Methods section.

n/a	Confirmed	
☐	☒	The exact sample size (*n*) for each experimental group/condition, given as a discrete number and unit of measurement
☐	☒	A statement on whether measurements were taken from distinct samples or whether the same sample was measured repeatedly
☐	☒	The statistical test(s) used AND whether they are one- or two-sided<br>*Only common tests should be described solely by name; describe more complex techniques in the Methods section.*
☐	☒	A description of all covariates tested
☐	☒	A description of any assumptions or corrections, such as tests of normality and adjustment for multiple comparisons
☐	☒	A full description of the statistical parameters including central tendency (e.g. means) or other basic estimates (e.g. regression coefficient) AND variation (e.g. standard deviation) or associated estimates of uncertainty (e.g. confidence intervals)
☐	☒	For null hypothesis testing, the test statistic (e.g. *F*, *t*, *r*) with confidence intervals, effect sizes, degrees of freedom and *P* value noted<br>*Give P values as exact values whenever suitable.*
☒	☐	For Bayesian analysis, information on the choice of priors and Markov chain Monte Carlo settings
☒	☐	For hierarchical and complex designs, identification of the appropriate level for tests and full reporting of outcomes
☒	☐	Estimates of effect sizes (e.g. Cohen's *d*, Pearson's *r*), indicating how they were calculated

Our web collection on statistics for biologists contains articles on many of the points above.

Software and code

Policy information about availability of computer code

Data collection	No software was used for data collection.
Data analysis	All custom codes used for data analyses are freely available from the following public repositories:<br>https://github.com/mcieslik-mctp/papy<br>https://github.com/mcieslik-mctp/hpseq<br>https://github.com/mcieslik-mctp/bootstrap-rnascape<br>https://github.com/mcieslik-mctp/codac<br>https://github.com/mcieslik-mctp/crisp<br>https://github.com/mcieslik-mctp/<br>https://github.com/mctp/<br>https://github.com/dovetail-genomics/dovetail_tools<br><br>Computational tools used:<br>GraphPad Prism 9 and in-built statistical tools<br>SAMtools (version 1.9 or 1.13)<br>PICARD Mark Duplicates (version 2.9.0)<br>HOMER (version v.4.10)<br>MACS2 (version 2.1.1.20160309)<br>bcl2fastq conversion software (v2.20)<br>BWA (version 0.7.17-r1198-dirty)<br>Pairtools (version 0.3.0)<br>EdgeR (version 3.34.1)<br>HTSeq-count (version 0.13.5)

deepTools (version 3.3.1)
ChipPeakAnno (version 3.0.0)
ChipSeeker (version 1.29.1)
R (version 3.6.0)
Cooler (version 0.8.11)
juicertools(version 1.22.01)
HiCExplorer (version 3.7)

For manuscripts utilizing custom algorithms or software that are central to the research but not yet described in published literature, software must be made available to editors and reviewers. We strongly encourage code deposition in a community repository (e.g. GitHub). See the Nature Research guidelines for submitting code & software for further information.

Data

Policy information about availability of data

All manuscripts must include a data availability statement. This statement should provide the following information, where applicable:

- Accession codes, unique identifiers, or web links for publicly available datasets
- A list of figures that have associated raw data
- A description of any restrictions on data availability

All raw data for the graphs, immunoblots, and gel electrophoresis figures are included in the Source Data or Supplementary Information. All materials are available from the authors upon reasonable request. All the raw next-generation sequencing, ATAC, ChIP, RNA, and HiChIP-seq data generated in this study have been deposited in the Gene Expression Omnibus (GEO) repository at NCBI (accession code GSE171592).

Field-specific reporting

Please select the one below that is the best fit for your research. If you are not sure, read the appropriate sections before making your selection.

☒ Life sciences ☐ Behavioural & social sciences ☐ Ecological, evolutionary & environmental sciences

For a reference copy of the document with all sections, see nature.com/documents/nr-reporting-summary-flat.pdf

Life sciences study design

All studies must disclose on these points even when the disclosure is negative.

Sample size	Sample sizes were empirically and statistically determined. For animal experiments, n=10-20 tumors were used for the pilot and efficacy studies. Using 20 tumors per treatment group, the statistical power to detect a 50% decrease in the mean tumor volume or metastatic burden in the treatment group is estimated to be 92.3% if the coefficient of variation (CV) is 40%. All in vitro experiments were performed with at least 3 technical replicates across two independent experiments. All samples sizes for various assays are listed in the Methods section or the figure legends.
Data exclusions	No data was excluded from the published publicly-available patient sequencing studies. For biological experiments, no data exclusions were made.
Replication	For all experiments, there are at least two independent biological repeats and multiple technical repeats in each. In all instances, all attempts at replicating the experiments produced similar results.
Randomization	For animal studies, mice were randomly assigned to treatment groups. For all other in vitro experiments, we used a common cell suspension to plate for both control and treatment groups.
Blinding	All histo-pathological evaluations of tissues and IHC/staining-based scoring for drug toxicity studies were carried out in a blinded manner by two independent pathologists. For all other experiments, the analyses did not require blinding as data quantification was carried out using instruments and automated workflows with no manual steps.

Reporting for specific materials, systems and methods

We require information from authors about some types of materials, experimental systems and methods used in many studies. Here, indicate whether each material, system or method listed is relevant to your study. If you are not sure if a list item applies to your research, read the appropriate section before selecting a response.

Materials & experimental systems

n/a	Involved in the study
☐	☒ Antibodies
☐	☒ Eukaryotic cell lines
☒	☐ Palaeontology and archaeology
☐	☒ Animals and other organisms
☒	☐ Human research participants
☒	☐ Clinical data
☒	☐ Dual use research of concern

Methods

n/a	Involved in the study
☐	☒ ChIP-seq
☒	☐ Flow cytometry
☒	☐ MRI-based neuroimaging

Antibodies

Antibodies used

Target antigen; Vendor; Catalog number; Lot number; Application; Note
BAF155 Cell Signaling Technology 11956S, Clone:D7F8S, Lot: 4, Western Blot, Co-IP 1:1000
SMARCA2/BRM Bethyl laboratories A301-016A, Lot: 1, Western Blot 1:1000
SMARCA4/BRG1 Cell Signaling Technoly 52251S, Lot: 1, Western Blot 1:1000
PBRM1 Bethyl laboratories A301-591A, Lot: 3, Western Blot 1:1000
BRD4 Cell Signaling Technology 13440S, Clone: E2A7X Western Blot 1:1000
BRD7 Proteintech 51009-2-AP Western Blot 1:1000
BRD9 Thermo Scientific PA5-113488, Lot: WE3273112, Western Blot 1:1000
Vinculin Millipore Sigma V9131 Western Blot 1:5000
VHL Thermo Fisher Scientific PA527322, Lot: UH2825110A, Western Blot 1:1000
AR Millipore Sigma 06-680, Lot: 3256650, Western Blot, Co-IP 1:1000
ERG Abcam ab92513 Western Blot, Co-IP 1:1000
FOXA1 Thermo Fisher Scientific PA5-27157 Western Blot, Co-IP 1:1000
c-Myc Cell Signaling Technology 5605S, Clone: D84C12, Western Blot 1:1000
PSA DAKO A0562, Lot: 00093790, Western Blot 1:4000
YY1 Diagenode C15410345 Western Blot 1:1000
MED1 Bethyl laboratories A300-793A Western Blot 1:1000
H3K27Me3 Diagenode C15410069 Western Blot 1:1000
H3K27Ac Cell Signaling Technology 8173, Clone: D5E4, Western Blot 1:1000
H3K4me3 Cell Signaling Technology 9751, Lot: 14, Clone: C42D8 Western Blot 1:1000
H3K4Me1 Abcam ab8895 Western Blot 1:1000
Cleaved PARP (Asp214) Cell Signaling Technology 9541, Clone: Asp214, Lot: 13, Western Blot 1:1000
SMARCA2/BRM Millipore sigma HPA029981 IHC 1:100
SMARCA4/BRG1 Abcam ab108318 IHC 1:100
AR Millipore Sigma 06-680 IHC 1:100
FOXA1 Thermo Fisher Scientific PA5-27157, Lot: VFS004672A, IHC 1:1000
ERG Cell Signaling Technology 97249S, Clone: A7L1G, Lot: 1, IHC 1:500, ChIP-seq 10 mg/7-8M cells
AR Millipore/Sigma 06-680 ChIP-seq 10 mg/7-8M cells
FOXA1 Thermo Fisher Scientific PA5-27157 ChIP-seq 10 mg/7-8M cells
H3K27Ac Abcam ab4729 ChIP-seq 10 mg/10M cells
CTCF Cell Signaling Technology 3418, Clone: D31H2, Lot: 4, HiChIP-seq 1.14 mg per IP
H3K4me3 Cell Signaling Technology 9751, Clone:C42D8, Lot: 14, HiChIP-seq 3.4 mg per IP
H3K27Ac Cell Signaling Technology 8173, Clone: D5E4, HiChIP-seq 0.4 mg per IP

Validation

All antibodies used in this study are from reputed commercial vendors and have been validated by the vendors (see website). QC data is directly available from all the vendor listed above and these antibodies have been commonly used in other publications. These details are included in the vendor web-links pasted below:
BAF155, https://www.cellsignal.com/products/primary-antibodies/smarcc1-baf155-d7f8s-rabbit-mab/11956
SMARCA2/BRM, https://www.bethyl.com/product/A301-016A/SMARCA2+BRM+Antibody
SMARCA2/BRG, https://www.sigmaaldrich.com/US/en/product/sigma/hpa029981
SMARCA4/BRG1, https://www.cellsignal.com/products/primary-antibodies/brg1-e9o6e-mouse-mab/52251
SMARCA4/BRG1, https://www.abcam.com/brg1-antibody-epr3912-ab108318.html
PBRM1, https://www.bethyl.com/product/A301-591A/PBRM1+Antibody
BRD4, https://www.cellsignal.com/products/primary-antibodies/brd4-e2a7x-rabbit-mab/13440
BRD7, https://www.ptgcn.com/products/BRD7-Antibody-51009-2-AP.htm
BRD9, https://www.thermofisher.com/antibody/product/BRD9-Antibody-Polyclonal/PA5-113488
Vinculin, https://www.sigmaaldrich.com/US/en/product/sigma/v9131
VHL, https://www.thermofisher.com/antibody/product/VHL-Antibody-Polyclonal/PA5-27322
AR, https://www.emdmillipore.com/US/en/product/Anti-Androgen-Receptor-Antibody,MM_NF-06-680
ERG, https://www.abcam.com/erg-antibody-epr3864-ab92513.html
ERG, https://www.cellsignal.com/products/primary-antibodies/erg-a7l1g-rabbit-mab/97249
FOXA1, https://www.thermofisher.com/antibody/product/FOXA1-Antibody-Polyclonal/PA5-27157
c-Myc, https://www.cellsignal.com/products/primary-antibodies/c-myc-d84c12-rabbit-mab/5605
YY1, https://www.diagenode.com/en/p/yy1-polyclonal-antibody-50-ug
MED1, https://www.bethyl.com/product/A300-793A/MED1+Antibody
H3K27Me3, https://www.diagenode.com/en/p/h3k27me3-polyclonal-antibody-classic-50-mg-34-ml
H3K27Ac, https://www.cellsignal.com/products/primary-antibodies/acetyl-histone-h3-lys27-d5e4-xp-rabbit-mab/8173

H3K27Ac, https://www.abcam.com/histone-h3-acetyl-k27-antibody-chip-grade-ab4729.html
H3K4Me1, https://www.abcam.com/Histone-H3-mono-methyl-K4-antibody-ChIP-Grade-ab8895.html?gclsrc=aw.ds|aw.ds&gclid=CjwKCAjwzt6LBhBeEiwAbPGOgUFEy8GIMv4Wyw4MgVMXASeZXmacJ3JbieaWOcgXasSovoW1pm9ypRoCEWMQAvD_BwE
H3K4Me3, https://www.cellsignal.com/products/primary-antibodies/tri-methyl-histone-h3-lys4-c42d8-rabbit-mab/9751
Cleaved PARP, https://www.cellsignal.com/products/primary-antibodies/cleaved-parp-asp214-antibody-human-specific/9541
CTCF, https://www.cellsignal.com/products/primary-antibodies/ctcf-d31h2-xp-rabbit-mab/3418

Eukaryotic cell lines

Policy information about cell lines

Cell line source(s)

Most cell lines were originally obtained from ATCC, DSMZ, ECACC, or internal stock. C4-2B cells were generously provided by Evan Keller, Ph.D. at the University of Michigan (who originally purchased them from ATCC), CWR-R1 cells, and a series of enzalutamide-resistant prostate cancer cell lines (LNCaP_Parental, LNCaP_EnzR, CWR-R1_Parental, CWR-R1_EnzR, VCaP_Parental and VCaP_EnzR) were generated in the lab of and generously provided by Donald Vander Griend, Ph.D. at the University of Illinois at Chicago. HeLa cells were purchased from ATCC. All the cells were genotyped to confirm their identity at the University of Michigan Sequencing Core and tested routinely for Mycoplasma contamination. Additionally, all the cell lines were genotyped every two months to confirm their identity. LNCaP, 22RV-1, CWR-R1, PC-3, and DU145 were grown in Gibco RPMI-1640 + 10% FBS (ThermoFisher, Waltham, MA). VCaP was grown in Gibco DMEM + 10% FBS (ThermoFisher, Waltham, MA).

Authentication

All cell lines were biweekly tested to be free of mycoplasma contamination and genotyped every month at the University of Michigan Sequencing Core using Profiler Plus (Applied Biosystems) and compared with corresponding short tandem repeat (STR) profiles in the ATCC database to authenticate their identity in culture between passages and experiments. In particular, we ensured that the STR profile of HeLa cells were always >90% similar to the original, early passage cells. Also, HeLa cells were cultured in a separate hood to avoid any cross-contamination.

Mycoplasma contamination

All cells were biweekly tested for mycoplasma contamination using the MycoAlert PLUS Mycoplasma Detection Kit (Lonza) and were found to be continually negative. More details are included in the Methods section

Commonly misidentified lines
(See ICLAC register)

None

Animals and other organisms

Policy information about studies involving animals; ARRIVE guidelines recommended for reporting animal research

Laboratory animals

Efficacy studies: 4-6 week old male CB17 severe combined immunodeficiency (SCID) mice were procured from the University of Michigan breeding colony. Pharmacokinetics study: 9-11 week old CD-1 male mice were used. All mice were maintained under the conditions of pathogen-free, 12 hours light/12 hours dark cycle, temperatures of 18-23°C, and 40-60% humidity.

Wild animals

No wild animals were used in the study.

Field-collected samples

No field collected samples were used in the study.

Ethics oversight

The Institutional Animal Care & Use Committee (IACUC) ensures that the highest animal welfare standards are maintained along with the conduct of accurate, valid scientific research through the supervision, coordination, training, guidance, and review of every project proposed to include the use of vertebrate animals at the University of Michigan.

Note that full information on the approval of the study protocol must also be provided in the manuscript.

ChIP-seq

Data deposition

☒ Confirm that both raw and final processed data have been deposited in a public database such as GEO.

☒ Confirm that you have deposited or provided access to graph files (e.g. BED files) for the called peaks.

Data access links
May remain private before publication.

We have deposited the raw as well as processed ATAC, RNA, ChIP and HiChIP sequencing files to the GEO superseries repository; accession #: GSE171592.

Files in database submission

GSE171592 Targeting SWI/SNF ATPases in enhancer-addicted prostate cancer Oct 28, 2021

GSE171584 Targeting SWI/SNF ATPases in enhancer-addicted prostate cancer [ATAC-seq] Oct 28, 2021 approved None
GSM5227748 VCaP_DMSO_R1_8h (ATAC-seq) Oct 28, 2021 approved BED
GSM5227749 VCaP_DMSO_R2_8h (ATAC-seq) Oct 28, 2021 approved BED
GSM5227750 VCaP_DMSO_R1_24h (ATAC-seq) Oct 28, 2021 approved BED
GSM5227751 VCaP_DMSO_R2_24h (ATAC-seq) Oct 28, 2021 approved BED
GSM5227752 VCaP_AU_R1_4h (ATAC-seq) Oct 28, 2021 approved BED
GSM5227753 VCaP_AU_R2_4h (ATAC-seq) Oct 28, 2021 approved BED
GSM5227754 VCaP_AU_R1_8h (ATAC-seq) Oct 28, 2021 approved BED

GSM5227755 VCaP_AU_R2_8h (ATAC-seq) Oct 28, 2021 approved BED
GSM5227756 VCaP_AU_R1_12h (ATAC-seq) Oct 28, 2021 approved BED
GSM5227757 VCaP_AU_R2_12h (ATAC-seq) Oct 28, 2021 approved BED
GSM5227758 VCaP_AU_R1_24h (ATAC-seq) Oct 28, 2021 approved BED
GSM5227759 VCaP_AU_R2_24h (ATAC-seq) Oct 28, 2021 approved BED
GSM5227760 VCaP_ZBC260_R1_8h (ATAC-seq) Oct 28, 2021 approved BED
GSM5227761 VCaP_ZBC260_R2_8h (ATAC-seq) Oct 28, 2021 approved BED
GSM5227762 VCaP_ZBC260_R1_24h (ATAC-seq) Oct 28, 2021 approved BED
GSM5227763 VCaP_ZBC260_R2_24h (ATAC-seq) Oct 28, 2021 approved BED
GSM5227764 LNCaP_DMSO_R1_24h (ATAC-seq) Oct 28, 2021 approved BED
GSM5227765 LNCaP_DMSO_R2_24h (ATAC-seq) Oct 28, 2021 approved BED
GSM5227766 LNCaP_AU_R1_12h (ATAC-seq) Oct 28, 2021 approved BED
GSM5227767 LNCaP_AU_R2_12h (ATAC-seq) Oct 28, 2021 approved BED
GSM5227768 LNCaP_AU_R1_24h (ATAC-seq) Oct 28, 2021 approved BED
GSM5227769 LNCaP_AU_R2_24h (ATAC-seq) Oct 28, 2021 approved BED
GSM5227770 LNCaP_sgNC+shNC_R1_72h (ATAC-seq) Oct 28, 2021 approved BED
GSM5227771 LNCaP_sgNC+shNC_R2_72h (ATAC-seq) Oct 28, 2021 approved BED
GSM5227772 LNCaP_sgSMARCA2_R1 (ATAC-seq) Oct 28, 2021 approved BED
GSM5227773 LNCaP_sgSMARCA2_R2 (ATAC-seq) Oct 28, 2021 approved BED
GSM5227774 LNCaP_sgSMARCA4_R1 (ATAC-seq) Oct 28, 2021 approved BED
GSM5227775 LNCaP_sgSMARCA4_R2 (ATAC-seq) Oct 28, 2021 approved BED
GSM5227776 LNCaP_sgSMARCA2+shSMARCA4_R1_72h (ATAC-seq) Oct 28, 2021 approved BED
GSM5227777 LNCaP_sgSMARCA2+shSMARCA4_R2_72h (ATAC-seq) Oct 28, 2021 approved BED
GSM5655507 AU-CBH-15330 @1uM for 0.5h-R1 Oct 28, 2021 approved BED
GSM5655508 AU-CBH-15330 @1uM for 0.5h-R2 Oct 28, 2021 approved BED
GSM5655509 AU-CBH-15330 @1uM for 1h-R1 Oct 28, 2021 approved BED
GSM5655510 AU-CBH-15330 @1uM for 1h-R2 Oct 28, 2021 approved BED

GSE171589 Targeting SWI/SNF ATPases in enhancer-addicted prostate cancer [ChIP-seq] Oct 28, 2021 approved None
GSM5228982 VCaP_DMSO_6h_AR (ChIP-seq) Oct 28, 2021 approved BED
GSM5228983 VCaP_AU_6h_AR (ChIP-seq) Oct 28, 2021 approved BED
GSM5228984 VCaP_DMSO_6h_FOXA1 (ChIP-seq) Oct 28, 2021 approved BED
GSM5228985 VCaP_AU_6h_FOXA1 (ChIP-seq) Oct 28, 2021 approved BED
GSM5228986 VCaP_DMSO_6h_ERG (ChIP-seq) Oct 28, 2021 approved BED
GSM5228987 VCaP_AU_6h_ERG (ChIP-seq) Oct 28, 2021 approved BED
GSM5228988 VCaP_DMSO_6h_CTCF (ChIP-seq) Oct 28, 2021 approved BED
GSM5228989 VCaP_AU_6h_CTCF (ChIP-seq) Oct 28, 2021 approved BED
GSM5228990 VCaP_DMSO_24h_H3K27Ac (ChIP-seq) Oct 28, 2021 approved BED
GSM5228991 VCaP_AU_24h_H3K27Ac (ChIP-seq) Oct 28, 2021 approved BED
GSM5228992 LNCaP_DMSO_6h_AR (ChIP-seq) Oct 28, 2021 approved BED
GSM5228993 LNCaP_AU_6h_AR (ChIP-seq) Oct 28, 2021 approved BED
GSM5228994 LNCaP_DMSO_6h_FOXA1 (ChIP-seq) Oct 28, 2021 approved BED
GSM5228995 LNCaP_AU_6h_FOXA1 (ChIP-seq) Oct 28, 2021 approved BED
GSM5228996 LNCaP_DMSO_6h_CTCF (ChIP-seq) Oct 28, 2021 approved BED
GSM5228997 LNCaP_AU_6h_CTCF (ChIP-seq) Oct 28, 2021 approved BED
GSM5228998 LNCaP_DMSO_24h_H3K27Ac (ChIP-seq) Oct 28, 2021 approved BED
GSM5228999 LNCaP_AU_24h_H3K27Ac (ChIP-seq) Oct 28, 2021 approved BED
GSM5655511 VCaP_AU1h_AR_Milli Oct 28, 2021 approved BED
GSM5655512 VCaP_AU1h_FOXA1-TFS Oct 28, 2021 approved BED
GSM5655513 VCaP_AU1h_H3K27Ac_abcam Oct 28, 2021 approved BED
GSM5655514 VCaP_AU2h_AR_Milli Oct 28, 2021 approved BED
GSM5655515 VCaP_AU2h_FOXA1-TFS Oct 28, 2021 approved BED
GSM5655516 VCaP_AU2h_H3K27Ac_abcam Oct 28, 2021 approved BED
GSM5655517 VCaP_AU4h_AR_Milli Oct 28, 2021 approved BED
GSM5655518 VCaP_AU4h_FOXA1-TFS Oct 28, 2021 approved BED
GSM5655519 VCaP_AU4h_H3K27Ac_abcam Oct 28, 2021 approved BED

GSE171523 Targeting SWI/SNF ATPases in enhancer-addicted prostate cancer [RNA-seq] Oct 28, 2021 approved None
GSM5226548 VCaP_DMSO_R1_24h (RNA-seq) Oct 28, 2021 approved TXT
GSM5226549 VCaP_DMSO_R2_24h (RNA-seq) Oct 28, 2021 approved TXT
GSM5226550 VCaP_AU_R1_4h (RNA-seq) Oct 28, 2021 approved TXT
GSM5226551 VCaP_AU_R2_4h (RNA-seq) Oct 28, 2021 approved TXT
GSM5226552 VCaP_AU_R1_8h (RNA-seq) Oct 28, 2021 approved TXT
GSM5226553 VCaP_AU_R2_8h (RNA-seq) Oct 28, 2021 approved TXT
GSM5226554 VCaP_AU_R1_12h (RNA-seq) Oct 28, 2021 approved TXT
GSM5226555 VCaP_AU_R2_12h (RNA-seq) Oct 28, 2021 approved TXT
GSM5226556 VCaP_AU_R1_24h (RNA-seq) Oct 28, 2021 approved TXT
GSM5226557 VCaP_AU_R2_24h (RNA-seq) Oct 28, 2021 approved TXT
GSM5226558 VCaP_ZBC260_R1_8h (RNA-seq) Oct 28, 2021 approved TXT
GSM5226559 VCaP_ZBC260_R2_8h (RNA-seq) Oct 28, 2021 approved TXT
GSM5226560 VCaP_ZBC260_R1_24h (RNA-seq) Oct 28, 2021 approved TXT
GSM5226561 VCaP_ZBC260_R2_24h (RNA-seq) Oct 28, 2021 approved TXT
GSM5226562 LNCaP_DMSO_R1_24h (RNA-seq) Oct 28, 2021 approved TXT
GSM5226563 LNCaP_DMSO_R2_24h (RNA-seq) Oct 28, 2021 approved TXT
GSM5226564 LNCaP_AU_R1_12h (RNA-seq) Oct 28, 2021 approved TXT
GSM5226565 LNCaP_AU_R2_12h (RNA-seq) Oct 28, 2021 approved TXT

GSM5226566 LNCaP_AU_R1_24h (RNA-seq) Oct 28, 2021 approved TXT
GSM5226567 LNCaP_AU_R2_24h (RNA-seq) Oct 28, 2021 approved TXT
GSM5226568 LAPC4_DMSO_R1_24h (RNA-seq) Oct 28, 2021 approved TXT
GSM5226569 LAPC4_DMSO_R2_24h (RNA-seq) Oct 28, 2021 approved TXT
GSM5226570 LAPC4_AU_R1_24h_0.1uM (RNA-seq) Oct 28, 2021 approved TXT
GSM5226571 LAPC4_AU_R2_24h_0.1uM (RNA-seq) Oct 28, 2021 approved TXT
GSM5226572 LAPC4_AU_R1_24h_1uM (RNA-seq) Oct 28, 2021 approved TXT
GSM5226573 LAPC4_AU_R2_24h_1uM (RNA-seq) Oct 28, 2021 approved TXT
GSM5655526 VCaP_DMSO_2h_1 Oct 28, 2021 approved TXT
GSM5655527 VCaP_DMSO_2h_2 Oct 28, 2021 approved TXT
GSM5655528 VCaP_AU-15330_1 uM_0.5h_1 Oct 28, 2021 approved TXT
GSM5655529 VCaP_AU-15330_1 uM_0.5h_2 Oct 28, 2021 approved TXT
GSM5655530 VCaP_AU-15330_1 uM_1h_1 Oct 28, 2021 approved TXT
GSM5655531 VCaP_AU-15330_1 uM_1h_2 Oct 28, 2021 approved TXT
GSM5655532 VCaP_AU-15330_1 uM_2h_1 Oct 28, 2021 approved TXT
GSM5655533 VCaP_AU-15330_1 uM_2h_2 Oct 28, 2021 approved TXT

GSE171591 Targeting enhancer addiction in prostate cancer by impeding chromatin accessibility [HiChIP-seq] Oct 28, 2021 approved None
GSM5229035 VCaP_DMSO_4h_H3K4me3 (HiChIP-seq) Oct 28, 2021 approved HIC
GSM5229036 VCaP_AU_4h_H3K4me4 (HiChIP-seq) Oct 28, 2021 approved HIC
GSM5229037 VCaP_DMSO_4h_H3K27Ac (HiChIP-seq) Oct 28, 2021 approved HIC
GSM5229038 VCaP_AU_4h_H3K27Ac (HiChIP-seq) Oct 28, 2021 approved HIC
GSM5229039 VCaP_DMSO_4h_CTCF (HiChIP-seq) Oct 28, 2021 approved HIC
GSM5229040 VCaP_AU_4h_CTCF (HiChIP-seq) Oct 28, 2021 approved HIC

Genome browser session (e.g. UCSC)

No longer applicable

Methodology

Replicates

Multiple biological as well as technical replicates are included.

Sequencing depth

ATACseq: Sequenced to 65-70M total reads, paired-end mode, 125bp read lengths. Over 97% of uniquely mapped reads.
ChIPseq: Sequenced to 50-70M total reads, paired-end mode, 125bp read lengths. Over 97% of uniquely mapped reads.
RNAseq: Sequenced to 25-30M total reads, paired-end mode, 125bp read lengths. Over 97% of uniquely mapped reads.
HiChIPseq: Sequenced to 200-225M total reads, paired-end mode, 125bp read lengths. Over 95% of uniquely mapped reads.

Antibodies

See Supplementary Table 2.

Peak calling parameters

MACS2 (Version 2.1.1.20160309) callpeak was used for performing peak calling with the following option: 'macs2 callpeak–call-summits–verbose 3 -g hs -f BAM -n OUT–qvalue 0.05'. For H3K27ac data, the broad option was used.

Data quality

FastQC was used to quality check the raw sequencing data using standard metrics and default thresholds.

Software

Using deepTools (version 3.3.1) bamCoverage, a coverage file (bigWig format) for each sample was created. The coverage was calculated as the number of reads per bin, where bins are short consecutive counting windows. While creating the coverage file, the data was normalized with respect to each library size. ChIP peak profile plots and read-density heat maps were generated using deepTools, and cistrome overlap analyses were carried out using the ChIPpeakAnno (version 3.0.0) or ChIPseeker (version 1.29.1) packages in R (version 3.6.0).
